# A novel method for single-cell data imputation using subspace regression

**DOI:** 10.1038/s41598-022-06500-4

**Published:** 2022-02-17

**Authors:** Duc Tran, Bang Tran, Hung Nguyen, Tin Nguyen

**Affiliations:** Department of Computer Science and Engineering, University of Nevada Reno, Reno, NV USA

**Keywords:** Data mining, Machine learning, Software, Statistical methods, Computational models

## Abstract

Recent advances in biochemistry and single-cell RNA sequencing (scRNA-seq) have allowed us to monitor the biological systems at the single-cell resolution. However, the low capture of mRNA material within individual cells often leads to inaccurate quantification of genetic material. Consequently, a significant amount of expression values are reported as missing, which are often referred to as dropouts. To overcome this challenge, we develop a novel imputation method, named single-cell Imputation via Subspace Regression (scISR), that can reliably recover the dropout values of scRNA-seq data. The scISR method first uses a hypothesis-testing technique to identify zero-valued entries that are most likely affected by dropout events and then estimates the dropout values using a subspace regression model. Our comprehensive evaluation using 25 publicly available scRNA-seq datasets and various simulation scenarios against five state-of-the-art methods demonstrates that scISR is better than other imputation methods in recovering scRNA-seq expression profiles via imputation. scISR consistently improves the quality of cluster analysis regardless of dropout rates, normalization techniques, and quantification schemes. The source code of scISR can be found on GitHub at https://github.com/duct317/scISR.

## Introduction

Bulk RNA sequencing (RNA-seq) has been the primary tool to study biological systems. Despite its popularity, bulk sequencing is unable to measure the heterogeneity inside complex tissues and cell-to-cell variability. Recently, advances in microfluidics and sequencing technologies have allowed us to measure the expression profiles of individual cells^1,2^. By allowing us to monitor the biological processes at the single-cell resolution, single-cell technologies (scRNA-seq) have enabled new research directions in genomics and transcriptomics research. These include various atlas projects^3,4^ aiming at building the references of all cell types in model organisms, transcriptome landscape visualization in complex tissues^5,6^, inference of cell developmental trajectories^7^, and predicting cell spatial position^8^. Such comprehensive decomposition of complex tissues holds enormous potential in both basic research and clinical applications^9,10^.

However, scRNA-seq data also comes with additional challenges^11^. One of the challenges is that sequencing mRNA within individual cells requires artificial amplification of DNA materials, leading to disproportionate distortions of relative transcript abundance and gene expression. Another outstanding challenge is the “dropout” phenomenon where a gene is highly expressed in one cell but does not express at all in another cell^12^. These dropout events usually occur due to the limitation of sequencing technologies when only a small amount of starting mRNA in individual cells can be captured, leading to low sequencing depth and failed amplification^13,14^. Since downstream analyses of scRNA-seq heavily rely on the accuracy of expression measurement, it is crucial to impute the zero expression values introduced by the dropout phenomenon and sequencing errors.

There have been a number of computational methods developed to impute single-cell data. These imputation methods can be classified into two categories: i) model-based methods and ii) model-free methods. Methods in the first category model the data using a mixture of two different distributions: one distribution represents the actual gene expression while the other accounts for the dropout events. Next, they estimate the model parameters and true expression values using the Expectation-Maximization (EM) algorithm^15^. Methods in this category include scImpute^16^, SAVER^17^, and BISCUIT^18^. scImpute uses a Gaussian distribution to model the actual expression and a Gamma distribution to model the dropout events. It estimates the model parameters and dropout values using the EM algorithm. Similarly, SAVER^17^ models read counts as a mixture of Poisson-Gamma distribution and then uses a Bayesian approach to estimate the true expression values. BISCUIT^18^ uses the Dirichlet process mixture model^19^ to perform data normalization, cells clustering, and dropouts imputation by simultaneously inferring clustering parameters, estimating technical variations (e.g., library size), and learning co-expression structures of each cluster.

Methods in the second category typically assume that expression values from the same dataset follow a certain data structure (manifold), whereas dropout events move the values away from the underlying structure. These methods use regression techniques to infer missing values from genes or cells that have similar expression patterns. Methods in this category include MAGIC^20^, DrImpute^21^, scScope^22^, DCA^23^, and DeepImpute^24^. MAGIC imputes zero values using heat diffusion^25^. The method first computes the affinity matrix between cells using a Gaussian kernel and then constructs the Markov transition matrix by normalizing and smoothing the computed affinity matrix. Finally, the method multiplies the exponentiated Markov matrix with the original data to obtain the imputed data. DrImpute^21^ uses a cluster ensemble strategy and consensus clustering to separate data into groups of similar cells and then imputes missing data by averaging expression values of similar cells. The other three methods (scScope, DeepImpute, and DCA) rely on deep neural networks to denoise the data and to impute the missing values. scScope uses a recurrent network layer to iteratively impute the zero-valued entries while DeepImpute randomly splits genes into subsets and builds sub-neural networks to estimate the missing values. DCA, on the other hand, extends the standard autoencoder to account for sparse count data by incorporating a noise model into their loss function.

The quality of data imputed by methods in the first category (model-based methods) is determined by the validity of the assumption of the distribution models. In addition, these methods usually require excessive computational power, which makes them slow in processing big datasets. Therefore, these statistical methods often rely on gene filtering steps to ease the computational burden. For methods in the second category (model-free approaches), their major drawbacks include i) relying on many parameters to fine-tune their models, which can lead to overfitting, and ii) tending to over-smoothen and remove the cell-to-cell stochasticity that represents meaningful biological variations in gene expression. More importantly, in addition to the limitations mentioned above, methods in both categories attempt to alter the expression of all zero-valued entries, including those not affected by dropout events. This may introduce false signals and further weaken their reliability.

Here we propose a new approach, scISR, that can reliably impute missing values from single-cell data. Our method consists of three modules. The first module performs hypothesis testing to identify the values that are likely to be impacted by the dropout events. By not altering the true zero values, we can avoid false imputations. The second module utilizes a data perturbation technique^26^ to automatically group genes with similar patterns into smaller groups. The third module imputes missing values affected by dropout events (identified in the first module) by learning the gene patterns in each gene group (identified in the second module). This strategy ensures that the true missing values are imputed by using only highly relevant information. In an extensive analysis using simulation and 25 real scRNA-seq datasets, we demonstrate that scISR improves the quality of clustering analysis of single-cell data while preserving the transcriptome landscape.

## Results

**Figure 1:**
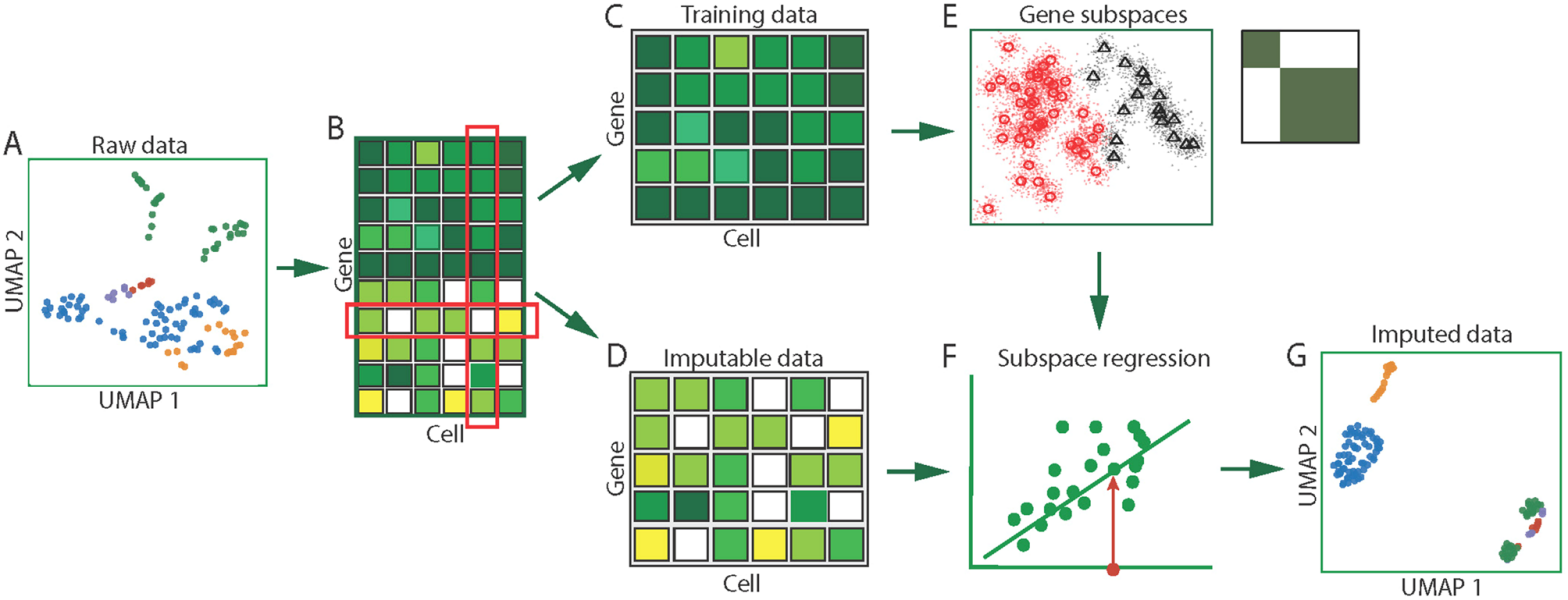
Single-cell Imputation using Subspace Regression (scISR). (**A**) Input data visualized in cell/sample space. (**B**) Hypergeometric test to determine whether each zero value is induced by dropout. Based on the computed p-values for each entry, we separate the original data into two sets of data: training data and imputable data. (**C**) Training data in which none of the values is induced by dropout events. (**D**) Imputable data in which each gene has at least one entry that is likely to be induced by dropout events. (**E**) Gene subspaces determined by perturbation clustering. We perturb the training data to discover the natural structure of the genes. Based on the pair-wise similarity between genes, we separate genes into groups that share similar patterns. (**F**) Subspace regression. We assign each gene in the imputable data to the closest subspace and then perform a generalized linear regression on the subspace to estimate the zero-valued entries that are impacted by dropouts. (**G**) Output expression matrix obtained by concatenating the training data and imputed data.

The schematic pipeline of scISR is shown in Fig. 1. The input is an expression matrix, in which rows represent genes/transcripts and columns represent cells/samples (Fig. 1A). The method consists of three modules. In the first module, we focus on identifying entries that are likely to be induced by dropouts (Fig. 1B). For this purpose, we perform a hypergeometric test on each zero-valued entry using the expression values in the corresponding gene-cell pair. An entry is imputable only if the p-value obtained from the test is significant. We then divide the data into two sets of data: (i) training data in which all values are trustworthy, i.e., no entry needs to be imputed (Fig. 1C), and (ii) imputable data in which each gene has at least one entry that needs to be imputed (Fig. 1D). In the second module, we aim at identifying similar gene groups (gene subspaces) in the training data that share similar expression patterns (Fig. 1E). For this purpose, we utilize the perturbation clustering we recently developed^59,26,27^. Finally, in the third module, we estimate the missing values in the imputable data using the identified gene subspaces (Fig. 1F). The method then merges the two matrices (training data and imputed data) and outputs a single matrix (Fig. 1G). The details of each module are provided in the “Methods” Section.

To assess the performance of scISR, we use both real scRNA-seq data and simulation. We compare scISR with five popular methods, MAGIC^20^, scImpute^16^, SAVER^17^, scScope^22^, and scGNN^28^. SAVER and scImpute are statistical approaches that impute the missing values using mixture models; MAGIC is a mathematical approach that relies on Markov transition to estimate the missing values. scScope uses a recurrent network layer to iteratively perform imputations on zero-valued entries of input scRNA-seq data. scGNN formulates and aggregates cell–cell relationships with graph neural networks and models heterogeneous gene expression patterns using a left-truncated mixture Gaussian model. scGNN uses the cell-cell relationships to impute the dropouts.

First, we apply the six methods on 25 real scRNA-seq datasets with known cell types. The cell labels are only used *a posteriori* to assess whether the imputation enhances the cell segregation, i.e., making the cell types more separable without drastically altering the transcriptome landscape. Second, we simulate 116 single-cell expression datasets whose values follow different distributions and dropout rates. We then apply the six imputation methods, scISR, MAGIC, scImpute, SAVER, scScope, and scGNN on the masked dataset to recover the missing values. Since we know exactly the missing entries and values, we can accurately assess the reliability of each method in terms of both sensitivity and specificity.

### scRNA-seq data and pre-processing

To assess the performance of the six imputation methods, we downloaded 25 publicly available scRNA-seq datasets available on NCBI, ArrayExpress, and Broad Institute Single Cell Portal (https://singlecell.broadinstitute.org/single_cell). The description of the datasets is shown in Table 1. The processed data of the first 15 datasets are also available at the Hemberg Lab’s website (https://hemberg-lab.github.io/scRNA.seq.datasets). There are 14 plate-based datasets and 11 droplet-based datasets. Among these, 12 datasets are with UMI, and 13 datasets are with read counts. There are 7 datasets without normalization while the remaining 18 datasets were already normalized by the data providers: 3 CPM-, 3 TPM-, 4 RPKM-, 4 FPKM-, and 4 RPM-normalized.

We analyzed the data with minimal additional pre-processing steps. For datasets with the range of values larger than 100, we rescale the data using log transformation (base 2). We also remove genes that do not contribute to the analysis, including: (i) genes expressed in less than two cells; and (ii) genes that have less than one percent of non-zero-valued entries. In all 25 single-cell datasets, the cell types are known. However, these cell labels are not provided to any of the imputation methods. They are only used *a posteriori* to assess the quality of the imputed data.

**Table 1:** Description of the 25 single-cell datasets used to assess the performance of imputation methods. The first three columns describe the name, accession ID, and tissue, while the following seven columns show the sequencing protocol, cell isolation technique, quantification scheme, normalized unit, dropout rate, number of cell types, and number of cells.

Dataset	Accession ID	Tissue	Sequencing protocol	Cell isolation	Quant. scheme	Norm. unit	Drop. rate	Class	Size
1. Fan^29^	GSE53386	Mouse Embryo	SUPeR-seq	Plate	Reads	FPKM	0.584	6	69
2. Treutlein^30^	GSE52583	Mouse Tissues	SMARTer	Plate	Reads	FPKM	0.902	5	80
3. Yan^31^	GSE36552	Human Embryo	Tang	Plate	Reads	RPKM	0.456	6	90
4. Goolam^32^	E-MTAB-3321	Mouse Embryo	Smart-Seq2	Plate	Reads	CPM	0.685	5	124
5. Deng^33^	GSE45719	Mouse Embryo	Smart-Seq	Plate	Reads	RPKM	0.605	6	268
6. Pollen^34^	SRP041736	Human Tissues	SMARTer	Plate	Reads	TPM	0.671	4	301
7. Darmanis^35^	GSE67835	Human Brain	SMARTer	Plate	Reads	CPM	0.808	9	466
8. Usoskin^36^	GSE59739	Mouse Brain	STRT-Seq	Plate	Reads	RPM	0.846	3	622
9. Camp^37^	GSE75140	Human Brain	SMARTer	Plate	Reads	FPKM	0.801	7	734
10. Klein^38^	GSE65525	Mouse Embryo	inDrop	Droplet	UMI	RPM	0.658	4	2717
11. Romanov^39^	GSE74672	Human Brain	SMARTer	Plate	UMI	-	0.878	7	2881
12. Segerstolpe^40^	E-MTAB-5061	Human Pancreas	Smart-Seq2	Plate	Reads	RPKM	0.823	15	3514
13. Manno^41^	GSE76381	Human Brain	STRT-Seq	Plate	UMI	-	0.86	56	4029
14. Marques^42^	GSE75330	Mouse Brain	Fluidigm C1	Plate	Reads	FPKM	0.891	13	5053
15. Baron^43^	GSE84133	Human Pancreas	inDrop	Droplet	UMI	TPM	0.906	14	8569
16. Sanderson^44^	SCP916	Mouse Tissues	10X Genomics	Droplet	Reads	-	0.764	11	12,648
17. Slyper	SCP345	Human Blood	10X Genomics	Droplet	UMI	-	0.956	8	13,316
18. Zilionis (Mouse)^45^	GSE127465	Mouse Lung	inDrop	Droplet	UMI	RPM	0.976	7	15,939
19. Tasic^46^	GSE115746	Mouse Visual Cortex	SMART-Seq	Plate	Reads	CPM	0.798	6	23,178
20. Zyl (Human)^47^	SCP780	Human Eye	inDrop	Droplet	UMI	-	0.913	19	24,023
21. Zilionis (Human)^45^	GSE127465	Human Lung	inDrop	Droplet	UMI	RPM	0.982	9	34,558
22. Wei^48^	SCP469	Human Synovium	10x Genomics	Droplet	UMI	TPM	0.915	9	41,565
23. Cao^49^	SCP454	Sea Squirt Embryos	10x Genomics	Droplet	UMI	-	0.821	7	90,579
24. Orozco^50^	GSE135133	Human Eye	10X Genomics	Droplet	UMI	RPKM	0.964	12	100,055
25. Darrah^51^	GSE139598	Human Blood	Drop-seq	Droplet	UMI	-	0.947	14	162,490

$^{1}$ UMI: Unique Molecular Identifier; CPM: Counts Per Million; RPM: Reads Per Million; RPKM: Reads Per Kilobase of transcript, per Million mapped reads; FPKM: Fragments Per Kilobase of transcript, per Million mapped reads.

### Cluster analysis of 25 scRNA-seq datasets

We use the known cell types of the 25 scRNA-seq datasets to assess whether the imputation helps separate cells of different types in cluster analysis. We compare scISR against MAGIC, scImpute, SAVER, scScope, and scGNN using three assessment metrics: Adjusted Rand Index (ARI)^52^, Jaccard Index (JI)^53^, and Purity Index (PI)^54^.

Given a dataset (raw data), we use k-means to cluster the cells using the true number of cell types *k* as the number of clusters. We calculate the Adjusted Rand Index (ARI)^52^ to compare k-means partitioning against the known cell labels. Rand Index (RI) measures the agreement between a given clustering and the ground truth. The ARI is the corrected-for-chance version of the RI. The ARI takes values from − 1 to 1, with the ARI expected to be 1 for a perfect agreement, and 0 for random partitionings. Next, we apply each of the six imputation methods to the raw data to obtain the imputed data. Again, we use k-means to partition the imputed data and calculate the ARI values using the true cell labels. We expect that by imputing the raw data, we obtain better data in which the cells of different types are more separable. Therefore, we assess the performance of each method by comparing the ARI of the imputed data against the ARI obtained from the raw data. We repeat the whole procedure for all 25 datasets to assess how well each imputation method performs.

**Figure 2:**
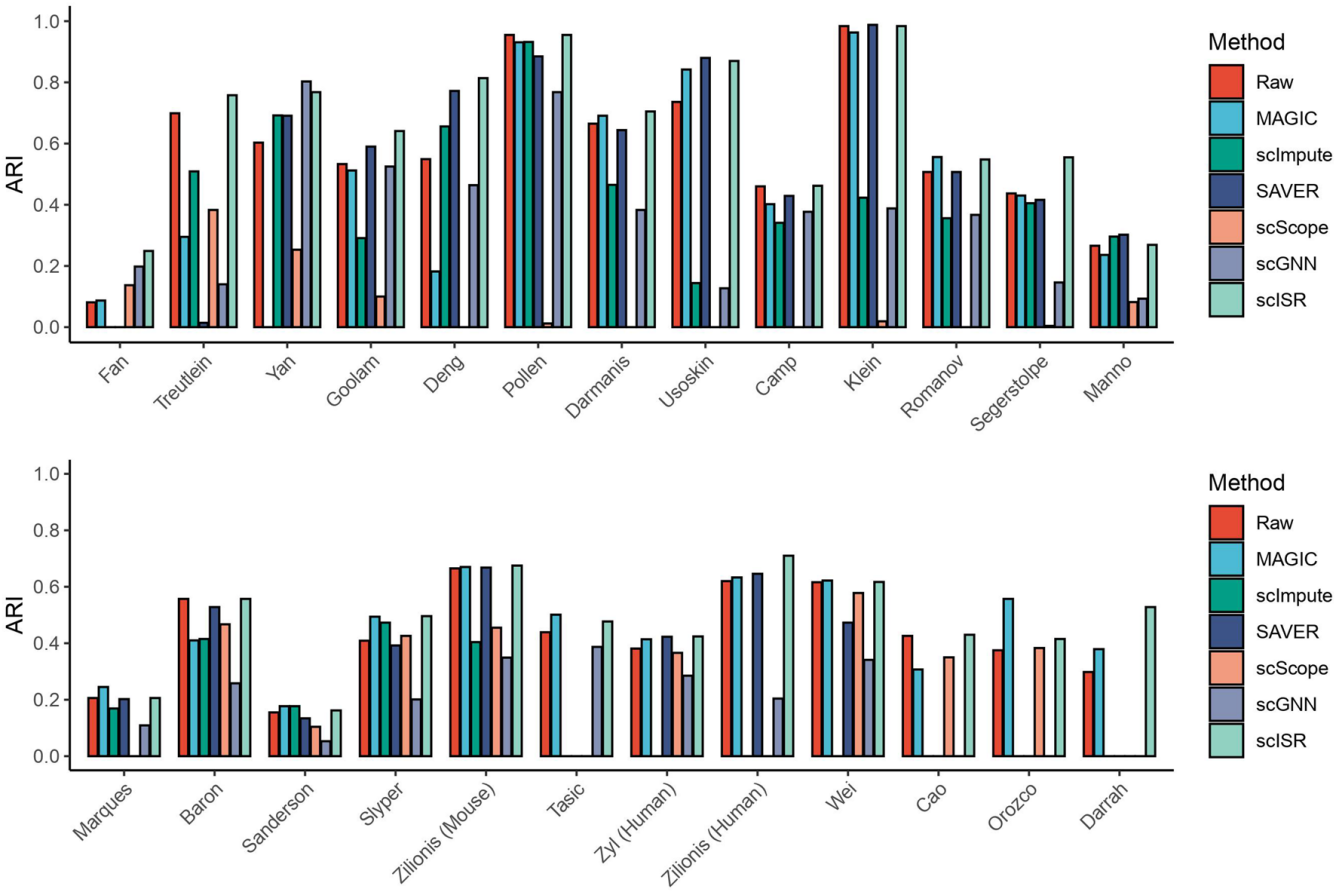
Adjusted Rand Index (ARI) obtained from raw and imputed data. The x-axis shows the names of the datasets while the y-axis shows ARI value of each method. scISR improves cluster analysis by having ARI values higher than those of the raw data in 21 out of 25 datasets.

Table 2 and Fig. 2 show the ARI values obtained for the 25 datasets. For each row, a cell of a method is highlighted in italic if the imputed ARI is higher than the raw ARI. The maximum memory permitted for each analysis was set to 100 GB of RAM. scISR and MAGIC are the only methods able to analyze all datasets. scImpute runs out of memory when analyzing datasets with 23,178 cells (Tasic) or larger. SAVER crashes when analyzing the Tasic dataset, and it runs out of memory when analyzing datasets with 90,579 cells (Cao) or larger. scScope runs out of memory when analyzing the biggest dataset (Darrah). scGNN ran out of memory when analyzing the datasets Cao, Orozco, and Darrah. We report the running time of imputation methods on 25 single-cell datasets in Supplementary Figure S1. Overall, scISR is the fastest method and can complete the imputation for the largest dataset (Darrah) in 50 minutes. For 25 real datasets, scISR is able to improve the ARI values 21 out of 25. The average ARI value of scISR is 0.571, which is the highest compared to those of raw data and data imputed by MAGIC, scImpute, SAVER, scScope, and scGNN (0.504, 0.461, 0.286, 0.423, 0.165, and 0.279, respectively). Overall, scISR increases the ARI values by 13.3% across all datasets. For the two datasets Zyl (Human) (24,023 cells) and Zilionis (Human) (34,558 cells), scISR increases the ARI values significantly (11.3% and 14.5%, respectively). For Orozco and Darrah datasets with more than 100,000 cells, scISR increases the ARI values by 13.6% and 77.2%, respectively. A one-sided Wilcoxon test also confirms that the ARI values of scISR are significantly higher than those of raw data ($p=3.2\times 10^{-5}$) and of other imputation methods ($p=9.8\times 10^{-6}$).

To perform a more comprehensive analysis, we also compare the methods using two other metrics: Jaccard Index (JI)^53^ and Purity Index (PI)^54^. The detailed results for each dataset and method are reported in Table 2 and Supplementary Tables S2–S3. Overall, scISR is the only method that has better clustering accuracy on average when comparing with using the raw data. The results are similar for analyses using JI and PI. Among all methods, scISR has the highest average JI values (Supplementary Table S2). Its average JI value is 0.531, compare to 0.468, 0.453, 0.276, 0.403, 0.243 and 0.273 of the raw data, MAGIC’s, scImpute’s, SAVER’s, scScope’s, and scGNN’s. A one-sided Wilcoxon test also confirms that the JI values of scISR are significantly higher than those of raw data ($p = 3.2\times 10^{-5}$) and of all other methods ($p = 4.8\times 10^{-5}$). Supplementary Table S3 shows the PI values obtained from raw and imputed data. It is the only method that has the average PI value higher than that of the raw data. All other methods have an average PI less than that of the raw data. scISR improves cluster analysis by having PI values higher than those of the raw data in 15 out of 25 datasets. A one-sided Wilcoxon test also confirms that the PI values of scISR are significantly higher than those of raw data ($p = 0.007$) and of all other methods ($p = 9.9\times 10^{-5}$). We also report the gene level normalized intra dispersion, which is the ratio between the intra-cell-type standard deviation and the gene’s standard deviation, in Supplementary Figure S2. The median dispersion of scISR is $3.6\times 10^{-3}$, which is much lower compared to $2\times 10^{-1}$, $1.1\times 10^{2}$, $2.4\times 10^{-1}$, $1.3\times 10^{-1}$, $2.3\times 10^{-2}$, and $5.4\times 10^{1}$ of raw data and data imputed by MAGIC, scImpute, SAVER, scScope and scGNN, respectively.

To further assess the performance of imputation methods, we perform an additional clustering analysis using Seurat^8^. This method can automatically determine the number of cell types from the input data. We first used Seurat to cluster the raw and imputed data of the 25 real scRNA-seq datasets. We then compared the clustering results against true cell types using Adjusted Rand Index (ARI). Supplementary Figure S3 and Table S4 show the ARI values obtained from the raw data and the data obtained from the six imputation methods. scISR is able to improve the cluster analysis in 14 out of 25 datasets. MAGIC, scImpute, SAVER, scScope, and scGNN improve the cluster analysis in 5, 3, 5, 4, and 5 datasets, respectively. The mean ARI value of scISR is 0.499, which is higher than the mean ARI values of all other methods (the mean ARI values for MAGIC, scImpute, SAVER, scScope, and scGNN are 0.315, 0.283, 0.324, 0.155, and 0.186, respectively). scISR is the only method that has the mean ARI higher than that of the raw data.

**Table 2:** Adjusted Rand Index (ARI) obtained from raw and imputed data. In each row, a cell is highlighted in  bold if the ARI value is higher than that of the raw data. scISR improves cluster analysis by having ARI values higher than those of the raw data in 21 out of 25 datasets. A one-sided Wilcoxon test also confirms that the ARI values of scISR are significantly higher than those of raw data ($p=3.2\times 10^{-5}$) and of all other methods ($p=9.8\times 10^{-6}$).

Dataset	Size	Raw	MAGIC	scImpute	SAVER	**scScope**	scGNN	scISR
Fan	69	0.081	**0.087**	0.000	0.000	**0.137**	**0.198**	**0.249**
Treutlein	80	0.699	0.295	0.509	0.014	0.383	0.140	**0.758**
Yan	90	0.603	0.000	**0.692**	**0.691**	0.253	**0.803**	**0.768**
Goolam	124	0.533	0.512	0.291	**0.590**	0.1	0.525	**0.641**
Deng	268	0.549	0.182	**0.656**	**0.772**	0	0.464	**0.814**
Pollen	301	0.955	0.931	0.932	0.885	0.012	0.768	0.955
Darmanis	466	0.665	**0.691**	0.465	0.644	0	0.383	**0.705**
Usoskin	622	0.736	**0.842**	0.144	**0.880**	0	0.127	**0.870**
Camp	734	0.460	0.402	0.341	0.429	0	0.377	**0.462**
Klein	2,717	0.984	0.963	0.423	**0.988**	0.019	0.388	0.984
Romanov	2,881	0.507	**0.556**	0.356	0.507	0	0.367	**0.548**
Segerstolpe	3,514	0.437	0.430	0.405	**0.576**	0.004	0.146	**0.555**
Manno	4,029	0.266	0.236	**0.296**	**0.302**	0.082	0.093	**0.269**
Marques	5,053	0.206	**0.245**	0.169	0.202	0	0.109	0.206
Baron	8,569	0.557	0.410	0.415	0.528	0.467	0.258	0.557
Sanderson	12,648	0.155	**0.177**	**0.177**	0.134	0.104	0.053	**0.162**
Slyper	13,316	0.409	**0.494**	**0.473**	0.392	**0.426**	0.201	**0.496**
Zilionis (Mouse)	15,939	0.665	**0.670**	0.404	**0.668**	0.455	0.349	**0.675**
Tasic	23,178	0.439	**0.501**	N/A	N/A	0	0.387	**0.477**
Zyl (Human)	24,023	0.381	**0.414**	N/A	**0.423**	0.366	0.285	**0.424**
Zilionis (Human)	34,558	0.620	**0.633**	N/A	**0.646**	0	0.204	**0.710**
Wei	41,565	0.616	**0.622**	N/A	0.473	0.578	0.341	**0.617**
Cao	90,579	0.426	0.307	N/A	N/A	0.35	N/A	**0.430**
Orozco	100,055	0.375	**0.557**	N/A	N/A	**0.383**	N/A	**0.415**
Darrah	162,490	0.298	**0.379**	N/A	N/A	N/A	N/A	**0.528**
Mean ARI		0.504	0.461	0.286	0.423	0.165	0.279	**0.571**

$^1$ N/A: Out of memory or error.

**Figure 3:**
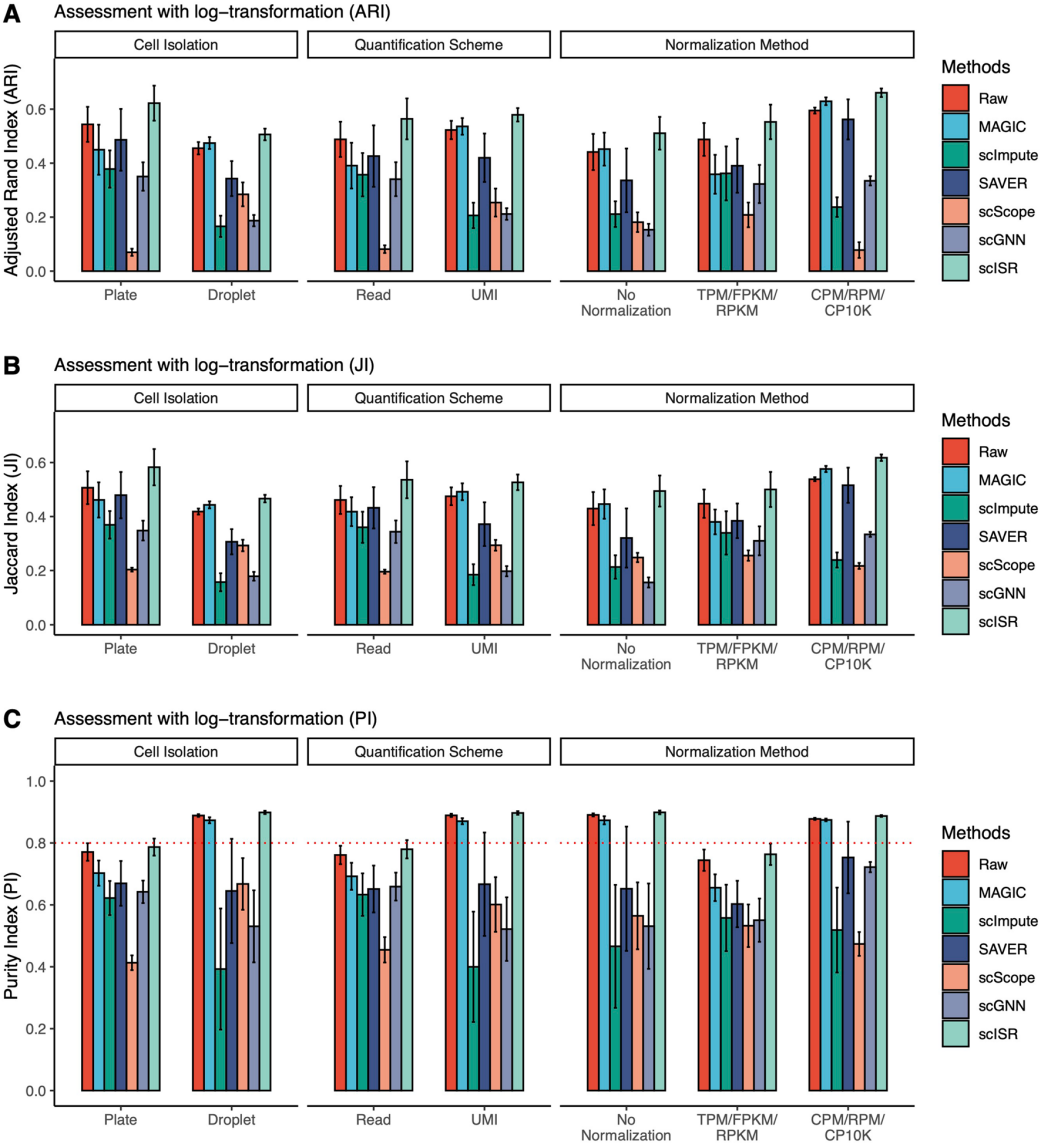
Assessment results of each imputation method with respect to cell isolation techniques, quantification schemes, or normalized units. The analysis is performed with a log transformation of the data. Panel (**A**) shows the results using Adjusted Rand Index (ARI), while panels (**B**) and (**C**) show the results using Jaccard Index (JI) and Purity Index (PI). scISR consistently outperforms other methods in every grouping by having the highest ARI, JI, and PI values.

Next, to assess the performance of each method with respect to different cell isolation techniques, quantitative schemes, and normalized units, we divide the datasets into multiple overlapping groups: (1) 14 plate-based and 11 droplet-based datasets; (2) 12 with UMI and 13 with read count; and (3) 7 without normalization, 11 with transcript length-normalization (RPKM/FPKM/TPM), and 7 with transcript-depth normalization (CPM/RPM). Fig. 2 shows the ARI values obtained for raw data and data imputed by four imputation methods. The ARI values of scISR are consistently higher than those of raw data and of other methods in each grouping. Interestingly, the ARI values of raw data are comparable across quantification schemes (UMI/read) but differ greatly across different normalization units (Fig. 3A). Well-known normalization techniques developed for bulk RNA-seq (RPKM/FPKM/TPM) improve raw data’s cluster analysis (better than no normalization), but they have apparent disadvantages compared to CPM/RPM. The ARI values of scISR follow the same trend but are always higher than those of raw data. Similarly, Figs. 3B and C show the JI and PI values obtained for the cluster analysis. Regardless of the assessment metrics, cluster analysis in conjunction with scISR has a notable advantage over other imputation methods.

To understand the impact of data scaling on the performance of the imputation methods, we also perform the same analysis without log transformation applied to the input data. Supplementary Figure S4 shows the overall results of the analysis while Supplementary Tables S5–S7 show the detailed results for each dataset and method. With the exception of scISR, a decrease in performance is observed for all imputation methods due to the dominance of genes with large values. This leads to a wider accuracy gap between scISR and other imputation methods.

### Preservation of the transcriptome landscape

The purpose of this analysis is to assess whether the imputation alters the transcriptome landscape. Preferably, life scientists impute the data in order to improve the quality of downstream analyses. At the same time, imputation should not completely change the data because of falsely introduced signals, leading to wrong or compromised findings. In the above sections, we have demonstrated that scISR significantly improves the quality of downstream analyses (e.g., cluster analysis). In this section, we will demonstrate that scISR preserves the transcriptome landscape of the data as well. For this purpose, we will visualize the transcriptome landscape of the raw and imputed data using t-SNE^55^ and UMAP^5^. We will also quantify the similarity between the imputed and original landscapes using the distance correlation index^56^.

First, we use t-SNE^55^ to generate the 2D transcriptome landscapes of the raw and imputed data. The 2D visualizations of the 25 datasets are shown in Supplementary Figures S6–S10. Overall, MAGIC, SAVER, and scISR produce landscapes that are similar to those of the raw data for every single dataset analyzed. The same cannot be said about scImpute, scScope, and scGNN. For the Manno dataset (the last row in Supplementary Figure S8), scImpute, scScope, and scGNN completely alter the landscape. scImpute tends to split cells into smaller groups while scScope and scGNN mix cells from different cell types together. This can be clearly observed in datasets such as Camp, Segerstolpe, Manno (Human).

To perform a more comprehensive analysis, we also generate the 2D transcriptome landscapes of the 25 datasets using UMAP^5^. The visualizations are shown in Supplementary Figures S11–S15. Again, except for scImpute, scScope, and scGNN, other methods preserve the landscape very well. For scImpute, scScope, and scGNN, the difference between the original and imputed landscape becomes more obvious in UMAP visualization.

To quantify the similarity between the imputed and original landscapes, we calculate the distance correlation index (*dCor*)^56^ for each imputed landscape generated by t-SNE and UMAP. Given *X* and *Y* as the 2D representations of the raw and imputed data, *dCor* is calculated as $dCor=\frac{dCov(X,Y)}{\sqrt{dVar(X)dVar(Y)}}$ where *dCov*(*X*, *Y*) is the distance covariance between *X* and *Y* while *dVar*(*X*) and *dVar*(*Y*) are distance variances of *X* and *Y*. Specifically, the method first calculates the pair-wise distances for *X* by computing the distance between each pair of cells, resulting in a square matrix. Second, it calculates the pair-wise distances for *Y*. Finally, it compares the two matrices using the formula described above to obtain the distance correlation. The *dCor* coefficient takes a value between 0 and 1, with the *dCor* is expected to be 1 for a perfect similarity. In our analysis, when we rotate the transcriptome landscape, *dCor* does not change. In contrast to Pearson correlation, this metric measures both the linear and nonlinear associations between *X* and *Y*^56^.

**Figure 4:**
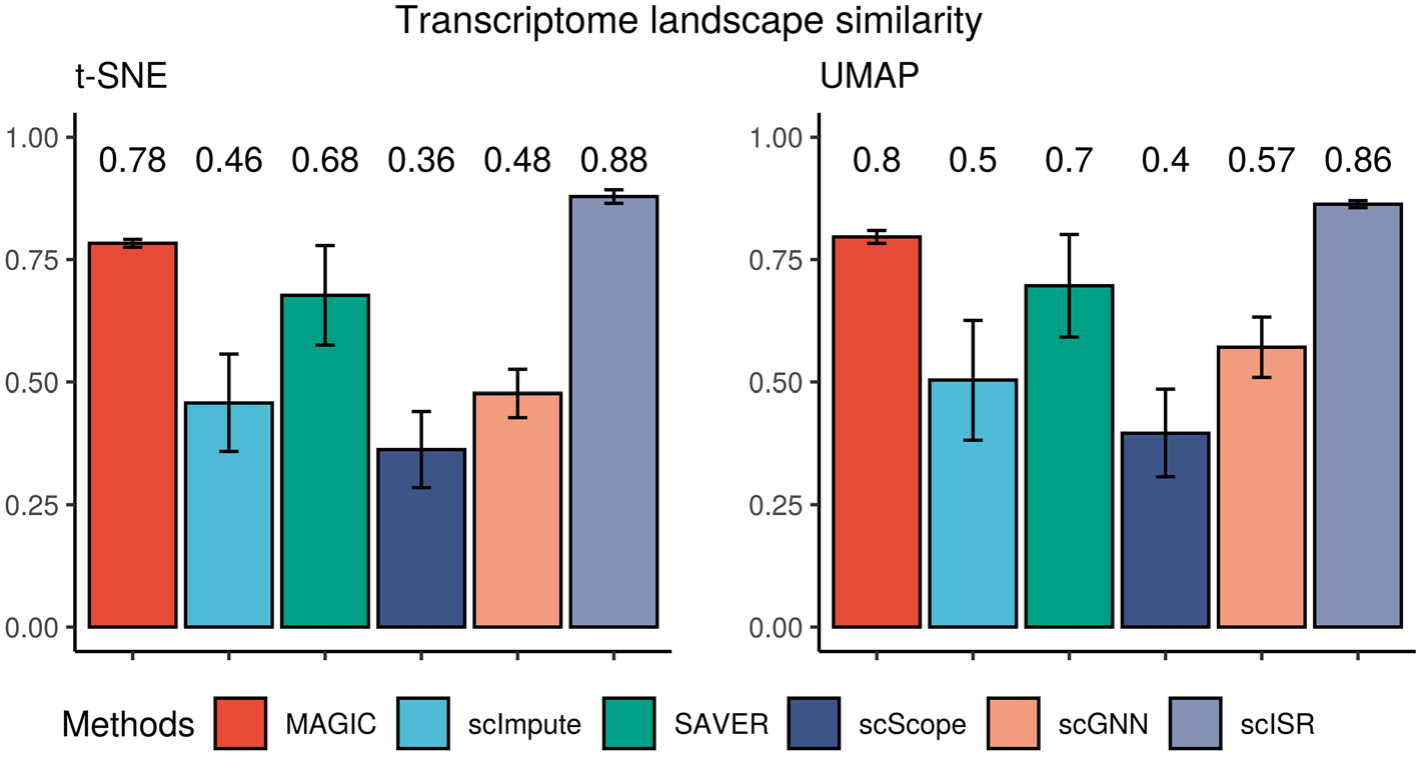
The distance correlation between raw data and imputed data using the first two components obtained from t-SNE and UMAP. Higher correlation values indicate more similarity between the imputed and original landscapes. Different colors represent different imputation methods. scISR has the highest mean correlation with the smallest variance. A one-sided Wilcoxon test indicates that the correlation values obtained from scISR are significantly higher than the rest ($p=3\times 10^{-9}$ and $2.8\times 10^{-7}$ for t-SNE and UMAP, respectively).

The *dCor* values are displayed in each panel in Supplementary Figures S6–S15. We also plot the *dCor* distributions in Fig. 4. In this figure, the left panel shows the values obtained from t-SNE while the right panel shows the values obtained from UMAP representations. The mean correlations using t-SNE for MAGIC, scImpute, SAVER, scScope, scGNN, and scISR are 0.78, 0.46, 0.68, 0.36, 0.48, and 0.88, respectively. The bar plot shows that scISR has the highest mean correlation, as well as the smallest variance. This demonstrates that scISR consistently preserves the transcriptome landscape of the datasets analyzed. MAGIC is the second-best method in this analysis. Using UMAP, scISR obtains a mean correlation of 0.86 compared to those of 0.8, 0.5, 0.7, 0.4, and 0.57, for MAGIC, scImpute, SAVER, scScope, and scGNN, respectively. A one-sided Wilcoxon test also confirms that the correlation values obtained from scISR are significantly higher than the rest ($p=3\times 10^{-9}$ and $2.8\times 10^{-7}$ for t-SNE and UMAP, respectively).

### Simulation studies

To present a comprehensive simulation analysis, we generate a total of 116 datasets in four different scenarios: (1) uniform dropout distribution, (2) normal dropout distribution, (3) highly correlated cell groups, and (4) Splatter-based simulation^57^.

In the first scenario, we generate 6 datasets by varying the number of cells from 100 to 10,000 and the number of genes from 300 to 10,000. The cells/genes combination setups are presented as follows: 100$\times$300, 1,000$\times$3,000, 3,000$\times$9,000, 5,000$\times$10,000, 7,000$\times$10,000, and 10,000$\times$10,000.

In each of the 6 datasets, the expression values follow a normal distribution $\mathcal {N}(\mu , \sigma )$. We set $\mu =1$ and $\sigma =0.15$. We slightly shift the mean of the cells and genes by adding a certain value to each group (− 1, 0, 1, 1.5 for cell groups and − 1, 0, 1 for gene groups) to create 4 different cell types and 3 gene groups – each cell type has an equal number of cells. We name this data as *complete data* and use the expression values as the ground truth for benchmarking. Next, we introduce the dropout events. We randomly select $40\%$ of the genes and consider those as genes that are impacted by dropout events. We randomly assign $30\%$ of the values of these genes to zero. We name this data as *masked data*.

The case studies for datasets with 100, 1000, and 10,000 cells are shown in Supplementary Figures S16, S17 and S18, respectively. In this simulation, dropout events clearly alter the cells’ transcriptome landscape, making it difficult to separate the 4 cell types. The ultimate goal of imputation is to infer the masked (dropout) values in order to recover the original transcriptome landscape and expression profile.

These case studies show that MAGIC imputes the missing values by smoothing the expression values. Many expression values, including non-zero-valued entries, were altered by MAGIC, making the landscape of the imputed data very different from those of both *complete* and *masked data*. scImpute improves the quality of the data but is still not able to separate some cell types. In addition, scImpute also alters the values of non-zero entries to make the data better fit into the assumed mixture model. SAVER further improves the transcriptome landscape and separates the 4 cell types. However, data imputed by SAVER does not entirely match with the *complete data*, in which many dropout values remain uncorrected many other dropout entries imputed with wrong values. scScope and scGNN oversmooth the imputed data such that it merges all the cells in four types together. The heatmaps clearly show that many expression values, including non-zero-valued entries, were altered by scScope and scGNN.

**Figure 5:**
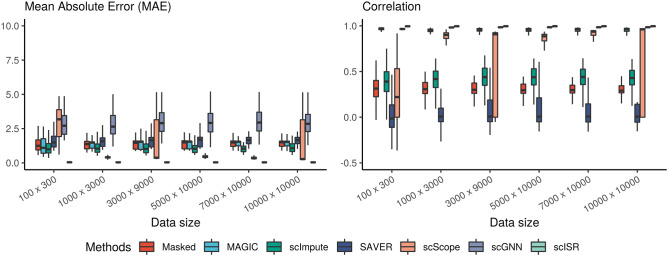
Assessment of MAGIC, scImpute, SAVER, scScope, scGNN, and scISR using simulation studies. Mean Absolute Error (MAE) and correlation coefficients were obtained by comparing imputed data with the complete data. In each analysis, scISR has smaller MAE values and higher correlation coefficients than other methods.

Using the true expression values of the complete data in all 6 datasets, we calculate the mean absolute error (MAE) and correlation between the imputed data and the ground truth for the genes that were impacted by dropout events. Figure 5 displays the mean absolute error (MAE) (left panel) and correlation values (right panel) for each method and each cell/gene combination. scISR is the best method in recovering the gene expression values with the smallest MAE and the highest correlation values.

In the second scenario, we generate in total 40 datasets resulted from the combination of 2 different dropout distributions: uniform and normal, 4 different dropout rates: 60%, 70%, 80%, and 90%, and 5 different sizes of data with the number of cells$\times$genes are: 1000$\times$3,000, 3,000$\times$9000, 5000$\times$10,000, 7000$\times$10,000, and 10,000$\times$10,000. Since scISR uses the hypergeometric test, which can be less accurate when the dropout probability does not follow a uniform distribution, we use this simulation to assess the stability of scISR when imputing data with different dropout distributions.

To generate datasets of a certain size (e.g., 1000$\times$3000), we first generate an expression matrix whose values follow a normal distribution $N(\mu , \sigma )$ where $\mu =1$ and $\sigma =0.15$. We then slightly shift the mean of the cells and genes by adding a certain value to each group (− 1, 0, 1, 1.5 for cell groups and − 1, 0, 1 for gene groups) to create 4 different cell types. We name this as *complete data*. Next, we randomly assign dropout values to the data in two different cases. In the first case, the dropout probability is uniformly distributed. In the second case, the dropout probability follows a normal distribution. For example, at 60% dropout rate, the dropout probability follows a distribution of *N*(0.6, 0.1). We then vary the dropout rate from 60% to 90%. We name the data with dropouts as *masked data*. Next, we impute the *masked data* using imputation methods to obtain the *imputed data*. Finally, to assess the performance of imputation methods, we compare the imputed data against the complete data using Mean Absolute Error (MAE) and correlation coefficients. The detailed results are presented in Supplementary Figure S19.

Overall, when the dropout probability is uniformly distributed, in all datasets, scISR is able to recover most of the dropout values, resulting in a median MAE close to zero and correlation coefficients close to one at any dropout rate. When the dropout probability is normally distributed, in all datasets, scISR still performs as well at 60 to 80% dropout. When the dropout rate is 90%, for the dataset of size 1,000$\times$3,000, scISR can recover only a part of the data (median MAE of approximately 2.11 compared to 3.65 of masked data). However, the results clearly show that the bigger the size of the data, the better scISR can recover the missing values. The reason for such improvement is that with the same dropout rate, larger datasets provide us with more data to learn from, leading to improved hypothesis testing (hypergeometric test) and prediction (linear regression). For datasets with 7,000 cells or more, the median MAE is close to zero for both uniform and normal distributions at any dropout rate. In summary, scISR (using hypergeometric test) performs well for large datasets with high dropout rates even when the dropout probability is not uniformly distributed. Moreover, scISR also outperforms other methods in recovering the missing data by having the lowest median MAE and highest median correlation.

In the next scenario, we generate 40 new simulated datasets, in which the cells of the same cell type have high correlation. We use the same combinations of number of cells, dropout rates, and dropout distributions as in the second scenario (see Supplementary Section 4.2 for the details of the simulation). Supplementary Figure S20 shows the results obtained from the 40 new simulated datasets. scISR outperforms other methods by having the lowest mean absolute errors and highest correlations in every analysis performed.

In the last scenario, we perform additional simulations with negative binomial distribution as the noise model using Splatter. We set the number of genes to 15,000 and the number of cell types to 3. We generated 30 datasets with different cell numbers: 5000, 10,000, 25,000, 50,000, 100,000 and 200,000. For each sample size, we varied the sparsity levels by adjusting the *dropout.mid* parameters (midpoint parameter for dropout logistic function of Splatter). We set *dropout.mid* to 2.5, 3, 3.5, 4, and 4.5, which led to sparsity levels of 84%, 87%, 89%, 91%, 93%, respectively.

We used the mean absolute error (MAE) values and correlation coefficients between the ground truth expression and imputed expression data to assess the performance of imputation methods. Supplementary Figure S22 shows the results, in which scISR and scScope are the only methods that can perform imputation on the biggest dataset. MAGIC, SAVER, scImpute, and scGNN cannot analyze datasets with are more than 100,000, 10,000, 10,000, and 50,000 cells, respectively. Overall, MAGIC, SAVER, scScope, and scGNN are unable to correctly recover the missing values, which leads to MAE values that are even higher than the masked data (data without imputation). scImpute has good results in small datasets but is unable to impute datasets with more than 10,000 cells. Even in datasets with 10,000 cells, scImpute returns errors when the dropout rate increases (91 and 93%). In contrast, scISR is able to improve the quality of the dropout data in all scenarios. We also report the running time for these simulation studies in Supplementary Figure S23. scISR and scScope are the only methods that can perform imputation on dataset with 200,000 cells. Both methods can analyze the largest dataset with 200,000 cells in approximately 100 to 200 minutes. Other methods either run out of memory or are unable to finish in a reasonable amount of time, which was set to one day.

## Conclusion

In this work, we introduced a new method to mitigate the effects of dropout events that frequently happen during the sequencing process of individual cells. The contribution is two-fold. First, by introducing a hypothesis testing procedure, we avoid altering true zero values. Second, the subspace regression provides a more accurate imputation by limiting the imputation to gene groups with similar expression patterns. We compared our approach with state-of-the-art methods using 25 real scRNA-seq datasets and 116 simulated datasets. We demonstrated that scISR outperforms other imputation methods in improving the quality of clustering analysis. At the same time, we also demonstrated that scISR preserves the transcriptome landscape of each dataset. Finally, we showed that scISR is robust against different dropout rates and distributions. We expect that scISR will be a very useful method that can improve the quality of single-cell data. The tool can be seamlessly incorporated into other single-cell analysis pipelines^60^.

## Methods

### Hyper-geometric testing (Module 1)

This section describes the first module in scISR which aims at determining whether each zero value observed is the result of dropouts. Our hypothesis is that dropout events happen randomly for a gene affected by this phenomenon. By treating each cell as an instance of the population, we also assume that the ratio of zero values (dropout probability) reported for each cell differ from each other. Using dropout probabilities from both genes and cells, we can calculate how likely each zero values is affected by dropout. If zero values caused by dropout are over-represented in a gene, we conclude that this gene is affected by dropout events.

Given a zero-valued entry, let us denote $p_1$ and $p_2$ as the probability of observing a zero value in the corresponding gene and cell, respectively. It follows that the chances of having zero values in a gene and in a cell follow binomial distributions denoted by *X*$\sim$ *Bin*(*n*, $p_{1}$) and *Y*$\sim$ *Bin*(*m*, $p_{2}$), respectively. *n* is the number of measured values for a gene, and *m* is the number of measured values for a cell. Under the null, we have $p=p_1=p_2$. If *X* and *Y* are independent, we have $X + Y$ $\sim$ *Bin(n+m*, *p*). Therefore, the conditional distribution of *X*, $P(X = x|X + Y = r)$, is a hyper-geometric where *x* is the number of observed zero values in the gene and *r* is the total number of observed zero values in the selected pair of gene and cell. The probability mass function of the hyper-geometric distribution can be written as follows:

1 $$\begin{aligned} \begin{aligned} P(X = x-1|X + Y = r-1)&= \frac{\genfrac(){0.0pt}0{n-1}{x-1}\genfrac(){0.0pt}0{m}{r - x}}{\genfrac(){0.0pt}0{n + m-1}{r-1}} \\ \end{aligned} \end{aligned}$$

Note that *X* and *Y* have an overlapping entry for each gene and cell pair. Therefore, we remove the overlapping entry from the hypergeometic formula by using: i) $n+m-1$ (instead of $n+m$) as the total number of observed values in the selected pair of gene and cell, ii) $n-1$ (instead of *n*) as the number of measured values for the gene, and iii) $x-1$ (instead of *x*) as the number of zero values observed in the gene.

Applying Eq. (1), we calculate the p-value for every zero-valued. We perform two different kinds of tests: an under-representation and over-representation analysis with a significance threshold set to 0.01 for both analyses. An entry with a significant p-value in the over-representation analysis is considered untrustworthy and should be imputed (imputable). An entry with a significant p-value in the under-representation analysis is considered trustworthy. An entry that is neither trustworthy nor untrustworthy should be left alone. These values will not be imputed, nor be used to impute other values. A gene is trustworthy if all of its entries are trustworthy. A gene is imputable when at least one of its values is imputable. Based on this hypothesis testing procedure, we obtain a set of genes that can be used for training (training data), and a set of genes that needed to be imputed (imputable data). See Supplementary Section 4.2, Figures S19, S21, and S24 for discussion about the robustness of scISR.

### Identifying gene subspaces (Module 2)

It is crucial that the missing values of a gene are inferred using related genes that share similar expression patterns. Therefore, this module aims at identifying gene groups of the training data, i.e., gene subspaces that share similar patterns. For this purpose, we utilize the perturbation clustering^26,27^ that we recently developed. The method is based on the observation that small changes in quantitative assays will be inherently presented even when there is no significant difference between genes. If distinct gene groups do exist, they must be stable with respect to small degrees of data perturbation. This is indeed the case, as we have demonstrated in our previous work that the pair-wise connectivity between data points of the same group is preserved when the data are perturbed.

**Figure 6:**
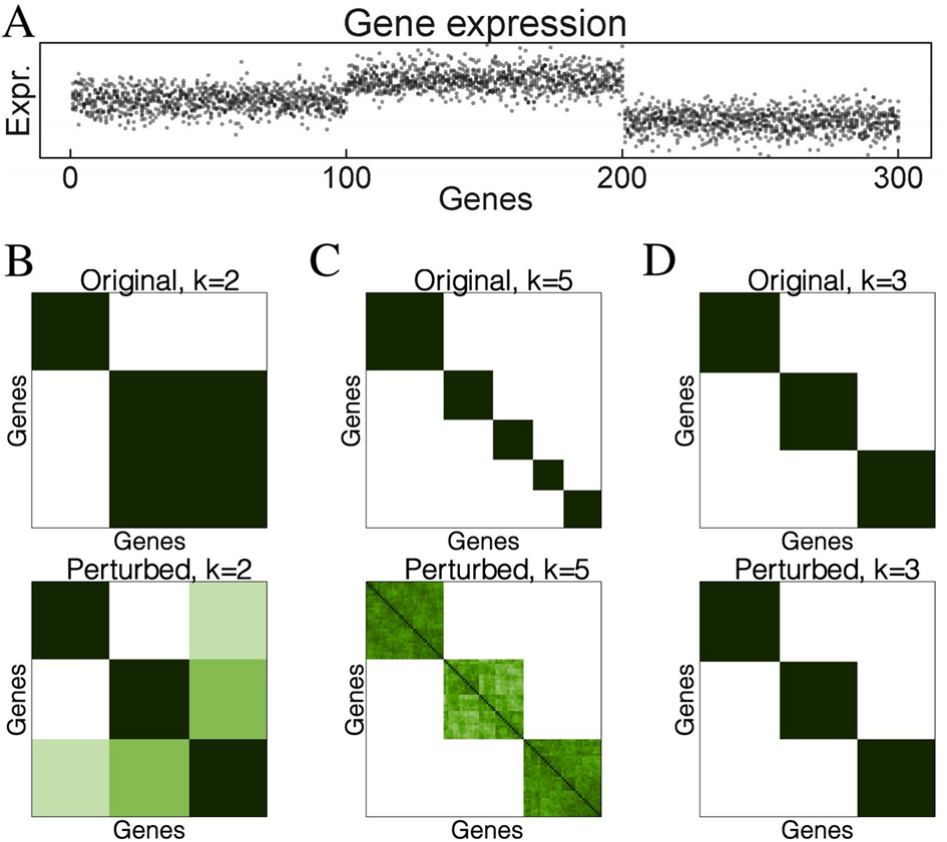
The resilience of pair-wise connectivity. (**A**) The dataset consists of three classes of genes: the first class has expression values of $\mathcal {N}(0,1)$, the second has expression values of $\mathcal {N}(1,1)$, and the third class has expression values of $\mathcal {N}(-1,1)$. (**B**) The original connectivity matrix (upper panel) and perturbed connectivity matrix (lower panel) for $k=2$. (**C**) The connectivity matrices for $k=5$. (**D**) The connectivity matrices for $k=3$. The perturbed connectivity matrices clearly reveal the true structure of the data.

We will describe this approach using an illustrative example shown in Fig. 6. In this simulated dataset, we have three distinct classes of genes in which the expressions of genes in each class are generated using a standard normal distribution. This distribution for the first class is $\mathcal {N}(0,1)$, for the second class is $\mathcal {N}(1,1)$ to simulate up-regulated genes, and for the third class is $\mathcal {N}(-1,1)$ to simulate down-regulated genes.

Assuming that we do not know the number of classes in this dataset, we set $k=2$ (number of clusters) and then partition the genes. The upper panel in Fig. 6B shows the connectivity between genes after clustering: green when they belong to the same cluster, and white otherwise. Note that two of the three true classes are wrongfully grouped together due to the wrong number of clusters. Now we repeatedly perturb the molecular measurements (by adding Gaussian noise) and partition the genes again (still with $k=2$). The lower panel in Fig. 6B shows the average connectivity between genes when the data is perturbed. The perturbed connectivity matrix suggests that the larger cluster is not stable. Similarly, the discordant connectivity in Fig. 6C states that the partitioning using $k=5$ is not correct either. The perturbed connectivity matrices (Fig. 6B, C) suggest that there are three distinct classes of genes. Finally, when we set $k=3$, the perturbed and original connectivity matrices are identical (Fig. 6D).

The perturbed connectivity matrices suggest that there are three distinct classes of genes. This demonstrates that for truly distinct gene groups the true connectivity between genes within each class is recovered when the data is perturbed, no matter how we set the value of *k*. This resilience of pair-wise connectivity occurs consistently regardless of the clustering algorithm being used (e.g., *k*-means, hierarchical clustering, or partitioning around medoids), or the distribution of the data. When there are no truly distinct subgroups, the connectivity is randomly distributed. When the number of true classes changes, the perturbed connectivity always reflects the true structure of the data.

To identify the optimal partitioning, we calculate the absolute difference between the original and the perturbed connectivity matrices and compute the empirical cumulative distribution functions of the entries of the difference matrix (CDF-DM). In the ideal case of perfectly stable clusters, the original and perturbed connectivity matrices are identical, yielding a difference matrix of 0s, a CDF-DM that jumps from 0 to 1 at the origin, and an area under the curve (AUC) of 1^59,26,27^. We choose the partitioning with the highest AUC and then partition the genes into subgroups that are strongly connected in those perturbation scenarios. We note that the idea of determining subspaces can be realized for both genes and cells simultaneously. We do not focus on such simultaneous clustering in this manuscript, but it is of great interest.

### Subspace regression (Module 3)

In the first module, we divide the genes into two sets: i) a set *I* in which all of the genes are likely to be affected by dropouts (imputable set), and ii) a set *T* that have accurate gene expression that does not need to impute (training set). In the second module, we segregate *T* into smaller groups of genes (gene subspaces) that share similar expression patterns. In this third module, we will impute dropout values in group *I* using a generalized linear regression model on gene subspaces.

Given a gene in the imputable set $g\in I$, we calculate the Euclidean distance between the gene to the centroid of each gene subspaces. Based on the calculated distances, we assign the gene to the closest subspace (with the smallest Euclidean distance). In order to impute dropout values in *g*, we train a generalized linear model using only highly-correlated genes within the assigned subspace in *T*. The linear regression process consists of two steps. The first step is to select genes from the training set that are highly correlated with the gene we need to impute. In the second step, we train the linear model using these highly correlated genes and then estimate the missing values^58^.

Denoting $y \subset g$ as the non-zero part of *g*, *S* as the gene subspace in *T* that *g* was assigned to, $\{s_i \in S\}$ are expression vectors of genes in *S*; and $\{x_i \subset t_i\}$ are the parts of $\{t_i\}$ that correspond with *y*. We calculate the Pearson correlation between *y* and $x_i$ and then select the 10 genes $\{t_{1},\ldots ,t_{10}\}$ in *T* with the highest correlation coefficients (see Supplementary Figure S5 for the discussion with regard to this parameter). We train a linear model in which $\{x_{1},\ldots ,x_{10}\}$ are the predictor variables and *y* is the outcome variable. In our implementation, we adopt the *lm* function that is available in the *stats* R package. Next, we use the trained linear model to estimate the missing values in $g \setminus y$, using $\{t_{1} \backslash {x_{1}},\ldots ,t_{10} \backslash x_{10}\}$ as the predictors, where $t_{i} \backslash {x_{i}}$ is the part of $t_{i}$ that does not belong to ${x_{i}}$. To avoid adding excessive weight to genes with high expression values, we always rescale the data to an acceptable range (default is [0,100]) using log transformation (base 2).

## Supplementary Information

Supplementary Information.

## Data Availability

All datasets analyzed in this manuscript are publicly available. The accession number for each dataset and its associated paper are reported in Table 1. The link to each dataset is available in Supplementary Table S1. The source code of the scISR package can be found on GitHub at https://github.com/duct317/scISR.
