# Supplementary material for: A novel method for single-cell data imputation using subspace regression
Source: Sci Rep. 2022 Feb 17;12:2697. doi: 10.1038/s41598-022-06500-4 (PMC8854597; doi:10.1038/s41598-022-06500-4)
Supplement: Supplementary file 1 — Supplementary Information. [file 41598_2022_6500_MOESM1_ESM.pdf]

A novel method for single-cell data imputation using subspace regression

Supplementary Information

Duc Tran[1], Bang Tran[1], Hung Nguyen[1], and Tin Nguyen[1,*]

[1] Department of Computer Science and Engineering, University of Nevada, Reno
[*] Corresponding: tinn@unr.edu

Table S1. Link to 25 single-cell datasets.

Dataset	Link
1. Fan	https://hemberg-lab.github.io/scRNA.seq.datasets/mouse/edev/#fan
2. Treutlein	https://hemberg-lab.github.io/scRNA.seq.datasets/mouse/tissues/
3. Yan	https://hemberg-lab.github.io/scRNA.seq.datasets/human/edev/
4. Goolam	https://hemberg-lab.github.io/scRNA.seq.datasets/mouse/edev/#goolam
5. Deng	https://hemberg-lab.github.io/scRNA.seq.datasets/mouse/edev/#deng
6. Pollen	https://hemberg-lab.github.io/scRNA.seq.datasets/human/tissues/#pollen
7. Darmanis	https://hemberg-lab.github.io/scRNA.seq.datasets/human/brain/#darmanis
8. Usoskin	https://hemberg-lab.github.io/scRNA.seq.datasets/mouse/brain/#usoskin
9. Camp	https://hemberg-lab.github.io/scRNA.seq.datasets/human/brain/
10. Klein	https://hemberg-lab.github.io/scRNA.seq.datasets/mouse/esc/
11. Romanov	https://hemberg-lab.github.io/scRNA.seq.datasets/mouse/brain/#romanov
12. Segerstolpe	https://hemberg-lab.github.io/scRNA.seq.datasets/human/pancreas/#segerstolpe
13. Manno	https://hemberg-lab.github.io/scRNA.seq.datasets/human/brain/#manno
14. Marques	https://hemberg-lab.github.io/scRNA.seq.datasets/mouse/brain/#marques
15. Baron	https://hemberg-lab.github.io/scRNA.seq.datasets/human/pancreas/
16. Sanderson	https://singlecell.broadinstitute.org/single_cell/study/SCP916/
17. Slyper	https://singlecell.broadinstitute.org/single_cell/study/SCP345/
18. Zilionis (Mouse)	https://www.ncbi.nlm.nih.gov/geo/query/acc.cgi?acc=GSE127465
19. Tasic	https://www.ncbi.nlm.nih.gov/geo/query/acc.cgi?acc=GSE115746
20. Zyl (Human)	https://singlecell.broadinstitute.org/single_cell/study/SCP780/
21. Zilionis (Human)	https://www.ncbi.nlm.nih.gov/geo/query/acc.cgi?acc=GSE127465
22. Wei	https://singlecell.broadinstitute.org/single_cell/study/SCP469/
23. Cao	https://singlecell.broadinstitute.org/single_cell/study/SCP454/
24. Orozco	https://singlecell.broadinstitute.org/single_cell/study/SCP484/
25. Darrah	https://www.ncbi.nlm.nih.gov/geo/query/acc.cgi?acc=GSE139598

1 Data and parameter setting

We downloaded 25 real scRNA-seq datasets available on NCBI[1], ArrayExpress[2], and Broad Institute Single Cell Portal (`https://singlecell.broadinstitute.org/single_cell`). The processed data of the first 15 datasets are also available at the Hemberg Lab's website (`https://hemberg-lab.github.io/scRNA.seq.datasets`). Table S1 shows the specific link to each of the 25 datasets.

We compared scISR with five imputation methods that are widely used for single-cell imputation: MAGIC[3], scImpute[4], SAVER[5], scScope[6], and scGNN[7]. The following packages were used in the analysis: i) MAGIC version 2.0.3 from GitHub (`https://github.com/KrishnaswamyLab/MAGIC`), ii) scImpute version 0.0.9 from CRAN, iii) SAVER version 1.1.1 from CRAN, iv) scScope version 0.1.5 from GitHub (`https://github.com/AltschulerWu-Lab/scScope`), and v) scGNN version 1.0.2 from GitHub (`https://github.com/juexinwang/scGNN`). We carefully followed the instruction and tutorial provided by the authors of each package. We executed the methods using default parameters and parameters that are suggested by the authors.

For cluster analysis, we used k-means (stats package, CRAN) in conjunction with PCA (irlba package, CRAN). We executed k-means using the first 20 principle components. In each dataset, the number of clusters is set to the true number of cell types. Since k-means often converges to local optima, we ran k-means multiple times in each analysis and choose the partition with the smallest square error. For small datasets (less than 10,000 cells), we set $nstart = 1000$ and $iter.max = 1000$ because running k-means with 1,000 initialization provides sufficient stability for small datasets. For large datasets (more than 10,000 cells), we set $nstart = 2000$ and $iter.max = 2000$. We used the known cell types to assess whether the imputation helps to separate cells of different types in cluster analysis. We compared the partitionings obtained from k-means against the true partitionings using Adjust Rand Index (ARI)[8], Jaccard Index (JI)[9], and Purity Index (PI)[10]. These metrics are implemented in the CRAN package mclust version 5.4.5[11].

We used two different methods to visualize the transcriptome landscapes of the raw and imputed data: t-SNE[12] and UMAP[13]. We first projected the data to the first 20 principal components and then used the two methods to visualize the data. In our analysis, we used the Rtsne version 0.15 and UMAP version 0.2.2.0, both downloaded from CRAN.

2 Clustering analysis on real data

Given a dataset (raw data), we use k-means to cluster the cells using the true number of cell types k as the number of clusters. We calculate the Jaccard Index (JI) and Purity Index (PI) to compare k-means partitioning against the known cell labels. Next, we apply each of the six imputation methods on the raw data to obtain the imputed data. Again, we use k-means to partition the imputed data and calculate the JI and PI values using the true cell labels. We repeat the whole procedure for each of the 25 datasets to assess how well each imputation method performs.

Table S2 shows the JI values obtained for the raw data and the data inferred by the six imputation methods. In this analysis, scISR also improves the clustering analysis in 21 out of 25 datasets by having the JI values higher than those of the raw data. Among all methods, scISR has the highest average JI values. Its average JI value is 0.531, compare to 0.468, 0.453, 0.276, 0.403, 0.243 and 0.273 of the raw data, MAGIC's, scImpute's, SAVER's, scScope's, and scGNN's. A Wilcoxon test also confirms that the JI values of scISR are significantly higher than those of raw data ($p = 3.2 \times 10^{-5}$) and of all other methods ($p = 4.6 \times 10^{-5}$).

Table S3 shows the PI values obtained from raw and imputed data. The results are similar to the analysis using ARI and JI. It is the only method that has the average PI value higher than that of the raw data. All other methods have an average PI less than that of the raw data. scISR improves cluster analysis by having PI values higher than those of the raw data in most datasets (15 out of 25). A Wilcoxon test also confirms that the PI values of scISR are significantly higher than those of raw data ($p = 0.006$) and of all other methods ($p = 8.8 \times 10^{-5}$).

Figure S1 shows the running time of imputation methods on 25 single-cell datasets. As seen in Figure S1, only scISR and MAGIC can analyze the Darrah dataset. scISR is the fastest method and can complete the imputation for this dataset in 50 minutes. MAGIC can analyze the Darrah dataset but it takes 170 minutes to finish the analysis. It takes scScope 350 minutes to analyze the second largest dataset (Orozco 100,000 cells). scImpute, SAVER, and scGNN cannot even analyze the three largest datasets.

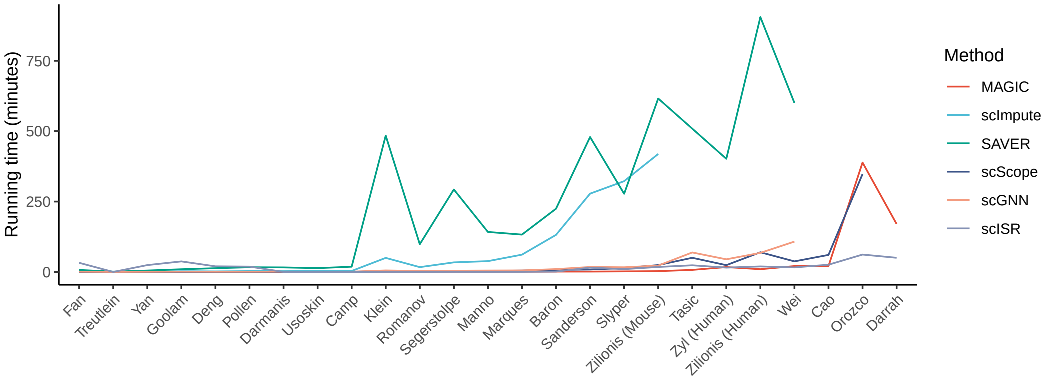

Figure S1. Running time of the six imputation methods on 25 real scRNA-seq datasets. scISR is the fastest and can impute the Darrah dataset in 50 minutes.

2.1 Normalized intra dispersion of imputed genes

For each gene, we calculated the ratio between the intra-cell-type standard deviation and the gene's standard deviation. The intra-cell-type standard deviation measures how similar the expression value of the cells for the underlying gene (cohesion). The ratio (between the intra-cell-type standard deviation and the gene's standard deviation) represents the normalized intra-cell-type standard deviation. We named this as intra dispersion. In general, we expect that with an improved data quality, the expression of cells of the same type are closer to one another compared to cells of different types. Therefore, we expect that a good imputation method would have the smallest intra dispersion. For each gene, we calculate the intra dispersion for the raw and imputed data: one value for raw data and 6 values for 6 imputation methods. Figure S2 shows the dispersion for each dataset. scISR has the smallest dispersion compared to raw data and data imputed by 5 other methods. Indeed, the median dispersion of scISR is 3.6×10^{-3} which is much lower compared to 2×10^{-1}, 1.1×10^{2}, 2.4×10^{-1}, 1.3×10^{-1}, 2.3×10^{-2}, and 5.4×10^{1} of raw data and data imputed by MAGIC, scImpute, SAVER, scScope and scGNN, respectively.

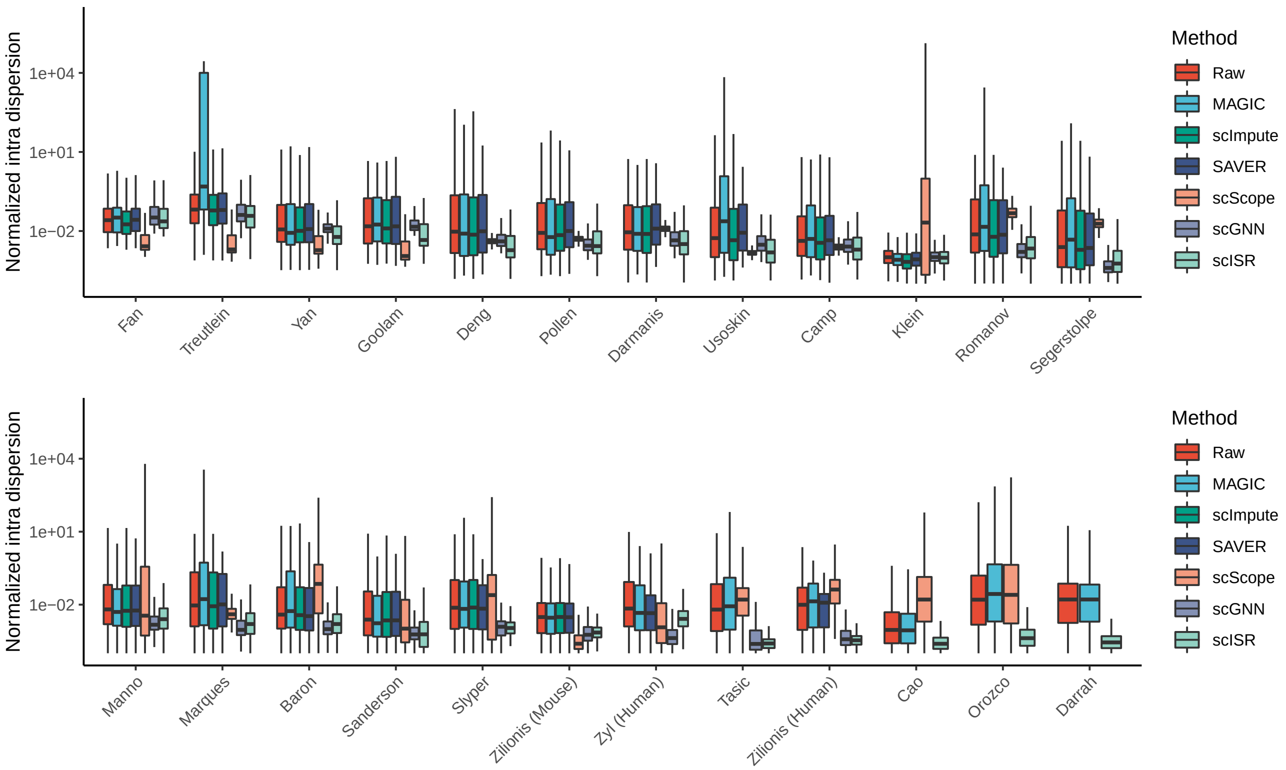

Figure S2. Distribution of the normalized intra dispersion for 25 real datasets. For each gene, we calculate the ratio between the intra-cell-type standard deviation and the gene's standard deviation (normalized intra dispersion). We repeat this calculation for all genes for raw and imputed data. The median dispersion of scISR is 3.6×10^{-3} which is much lower compared to 2×10^{-1}, 1.1×10^{2}, 2.4×10^{-1}, 1.3×10^{-1}, 2.3×10^{-2}, and 5.4×10^{1} of raw data and data imputed by MAGIC, scImpute, SAVER, scScope and scGNN, respectively.

Table S2. Jaccard Index (JI) obtained from raw and imputed data. In each row, a cell value is bold if the JI value is higher than that of the raw data. scISR improves cluster analysis by having JI values higher than those of the raw data in 21 out of 25 datasets. A Wilcoxon test also confirms that the JI values of scISR are significantly higher than those of raw data ($p = 3.2 \times 10^{-5}$) and of all other methods ($p = 4.8 \times 10^{-5}$).

Dataset	Size	Raw	MAGIC	scImpute	SAVER	scScope	scGNN	scISR
**Fan**	69	0.195	**0.223**	0.156	0.177	0.172	**0.226**	**0.261**
**Treutlein**	80	0.673	0.433	0.482	0.316	0.377	0.296	**0.727**
**Yan**	90	0.524	0.194	**0.612**	**0.608**	0.245	**0.734**	**0.695**
**Goolam**	124	0.513	0.496	0.359	**0.607**	0.195	0.506	**0.643**
**Deng**	268	0.524	0.333	**0.629**	**0.739**	0.293	0.446	**0.780**
**Pollen**	301	0.923	0.885	0.886	0.816	0.112	0.656	**0.924**
**Darmanis**	466	0.563	**0.594**	0.379	0.541	0.169	0.319	**0.606**
**Usoskin**	622	0.679	**0.795**	0.264	**0.840**	0.273	0.249	**0.828**
**Camp**	734	0.395	0.368	0.306	0.390	0.211	0.359	**0.398**
**Klein**	2,717	0.977	0.948	0.430	**0.983**	0.275	0.386	0.977
**Romanov**	2,881	0.451	**0.505**	0.316	**0.466**	0.249	0.326	**0.485**
**Segerstolpe**	3,514	0.363	0.356	0.330	0.330	0.228	0.137	**0.464**
**Manno**	4,029	0.167	0.147	**0.187**	**0.191**	0.056	0.061	**0.168**
**Marques**	5,053	0.168	**0.199**	0.149	**0.170**	0.106	0.107	0.168
**Baron**	8,569	0.445	0.324	0.326	0.418	0.374	0.207	0.445
**Sanderson**	12,648	0.243	**0.277**	**0.273**	0.225	0.2	0.120	**0.256**
**Slyper**	13,316	0.393	**0.476**	**0.458**	0.381	**0.427**	0.232	**0.478**
**Zilionis (Mouse)**	15,939	0.601	**0.607**	0.354	**0.602**	0.409	0.337	**0.610**
**Tasic**	23,178	0.431	**0.490**	N/A	N/A	0.134	0.389	**0.520**
**Zyl**	24,023	0.287	**0.315**	N/A	**0.324**	0.281	0.215	**0.323**
**Zilionis (Human)**	34,558	0.530	**0.546**	N/A	**0.556**	0.09	0.211	**0.633**
**Wei**	41,565	0.535	**0.541**	N/A	0.400	0.499	0.317	0.535
**Cao**	90,579	0.374	0.305	N/A	N/A	0.326	N/A	**0.379**
**Orozco**	100,055	0.375	**0.533**	N/A	N/A	0.364	N/A	**0.395**
**Darrah**	162,490	0.369	**0.446**	N/A	N/A	N/A	N/A	**0.589**
**Mean**		0.468	0.453	0.276	0.403	0.243	0.273	**0.531**

[1] N/A: Out of memory or error.

Table S3. Purity Index (PI) obtained from raw and imputed data. scISR improves cluster analysis by having PI values higher than those of the raw data in 15 out of 25 datasets. A Wilcoxon test also confirms that the PI values of scISR are significantly higher than those of raw data ($p = 0.007$) and of all other methods ($p = 9.9 \times 10^{-5}$).

Dataset	Size	Raw	MAGIC	scImpute	SAVER	scScope	scGNN	scISR
**Fan**	69	0.485	0.424	0.379	0.379	**0.5**	**0.500**	**0.545**
**Treutlein**	80	0.800	0.662	**0.825**	0.538	0.738	0.550	**0.838**
**Yan**	90	0.811	0.356	0.811	**0.833**	0.567	**0.867**	**0.844**
**Goolam**	124	0.823	0.815	0.758	0.774	0.597	**0.863**	0.823
**Deng**	268	0.806	0.660	0.795	0.795	0.507	0.795	**0.840**
**Pollen**	301	0.963	0.920	0.924	0.870	0.236	0.857	0.963
**Darmanis**	466	0.841	0.820	0.702	0.830	0.283	0.655	**0.848**
**Usoskin**	622	0.830	**0.879**	0.524	**0.929**	0.378	0.505	**0.929**
**Camp**	734	0.738	0.651	0.614	0.655	0.307	0.598	**0.740**
**Klein**	2,717	0.991	0.979	0.650	**0.994**	0.351	0.633	0.991
**Romanov**	2,881	0.845	0.800	0.760	0.817	0.35	0.732	**0.861**
**Segerstolpe**	3,514	0.840	0.822	0.773	**0.869**	0.377	0.666	**0.847**
**Manno**	4,029	0.506	0.463	**0.509**	0.467	0.266	0.282	0.506
**Marques**	5,053	0.445	**0.479**	**0.446**	0.440	0.19	0.345	0.445
**Baron**	8,569	0.947	0.856	0.833	0.935	0.888	0.703	0.947
**Sanderson**	12,648	0.936	**0.964**	**0.944**	**0.957**	0.858	0.877	**0.958**
**Slyper**	13,316	0.907	0.906	0.895	0.882	0.867	0.762	**0.917**
**Zilionis (Mouse)**	15,939	0.976	0.970	0.887	0.976	0.853	0.762	0.973
**Tasic**	23,178	0.912	0.907	N/A	N/A	0.485	0.874	0.856
**Zyl**	24,023	0.861	**0.878**	N/A	**0.863**	0.787	0.780	**0.875**
**Zilionis (Human)**	34,558	0.918	**0.930**	N/A	**0.946**	0.37	0.663	**0.920**
**Wei**	41,565	0.768	**0.773**	N/A	0.719	0.748	0.559	0.768
**Cao**	90,579	0.776	0.643	N/A	N/A	0.712	N/A	0.761
**Orozco**	100,055	0.918	**0.966**	N/A	N/A	0.911	N/A	**0.928**
**Darrah**	162,490	0.924	0.921	N/A	N/A	N/A	N/A	**0.942**
**Mean**		0.823	0.778	0.521	0.659	0.525	0.593	**0.835**

[1] N/A: Out of memory or error.

2.2 Assessment results using Seurat on real data

To further assess the performance of imputation methods, we perform an additional clustering analysis using Seurat[14]. This method can automatically determine the number of cell types from the input data. We first used Seurat to cluster the raw and imputed data of the 25 real scRNA-seq datasets. We then compared the clustering results against true cell types using Adjusted Rand Index (ARI). Figure S3 and Table S4 show the ARI values obtained from the raw data and the data obtained from the six imputation methods. scISR is able to improve the cluster analysis in 14 out of 25 datasets. MAGIC, scImpute, SAVER, scScope, and scGNN improve the cluster analysis in 5, 3, 5, 4, and 5 datasets, respectively. The mean ARI value of scISR is 0.499 which is higher than the mean ARI values of all other methods (the mean ARI values for MAGIC, scImpute, SAVER, scScope, and scGNN are 0.315, 0.283, 0.324, 0.155, and 0.186, respectively). scISR is the only method that has mean ARI higher than that of the raw data.

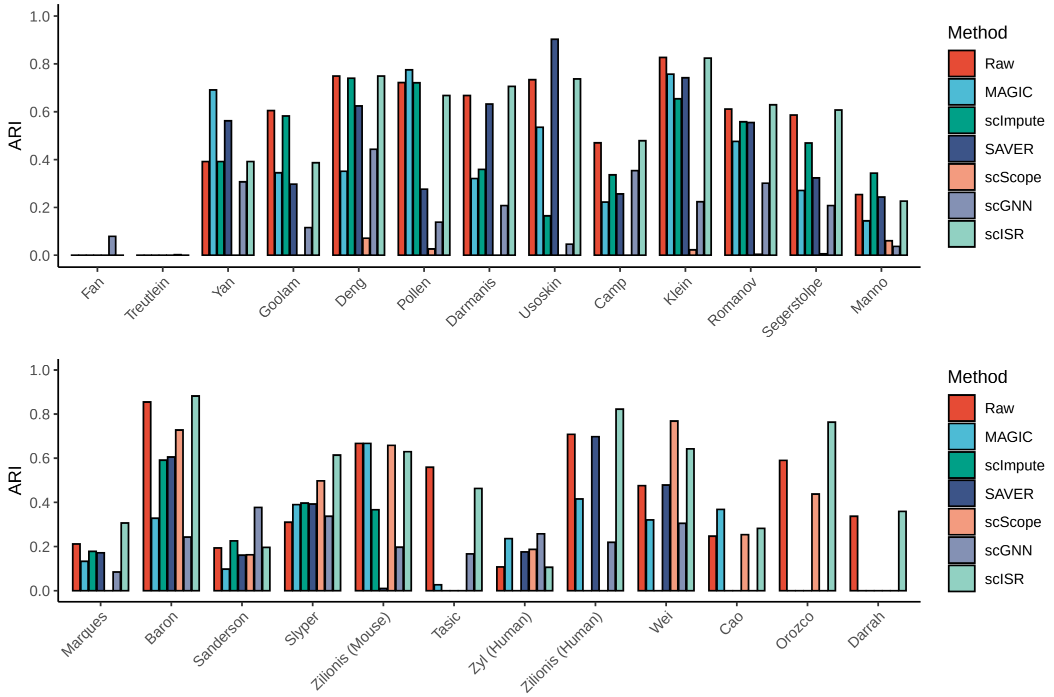

Figure S3. Adjusted Rand Index (ARI) obtained from raw and imputed data using Seurat as the clustering method. The x-axis shows the names of the datasets while the y-axis shows ARI value of each method.

Table S4. Adjusted Rand Index (ARI) obtained from raw and imputed data using Seurat as the clustering method. scISR improves cluster analysis by having ARI values higher than those of the raw data in 14 out of 25 datasets.

Dataset	Size	Raw	MAGIC	scImpute	SAVER	scScope	scGNN	scISR
**Fan**	69	0.000	0	0	0	0	**0.079**	0
**Treutlein**	80	0.000	0	0	0	0	**0.003**	0
**Yan**	90	0.392	**0.691**	0.392	**0.562**	0	0.307	0.392
**Goolam**	124	0.605	0.345	0.582	0.297	0	0.116	0.387
**Deng**	268	0.749	0.351	0.74	0.624	0.071	0.443	0.749
**Pollen**	301	0.722	**0.775**	0.721	0.276	0.026	0.138	0.668
**Darmanis**	466	0.668	0.321	0.359	0.632	0	0.208	**0.706**
**Usoskin**	622	0.734	0.535	0.165	**0.903**	0	0.046	**0.737**
**Camp**	734	0.470	0.222	0.336	0.256	0	0.354	**0.479**
**Klein**	2,717	0.827	0.757	0.654	0.742	0.023	0.224	0.824
**Romanov**	2,881	0.611	0.476	0.558	0.555	0.004	0.301	**0.629**
**Segerstolpe**	3,514	0.586	0.271	0.469	0.323	0.006	0.208	**0.607**
**Manno**	4,029	0.254	0.144	**0.343**	0.243	0.061	0.037	0.226
**Marques**	5,053	0.212	0.133	0.178	0.172	0	0.085	**0.307**
**Baron**	8,569	0.855	0.328	0.591	0.606	0.728	0.243	**0.882**
**Sanderson**	12,648	0.194	0.098	**0.226**	0.161	0.163	**0.377**	**0.196**
**Slyper**	13,316	0.310	**0.39**	**0.397**	**0.393**	**0.498**	**0.337**	**0.614**
**Zilionis (Mouse)**	15,939	0.667	0.667	0.367	0.01	0.658	0.197	0.63
**Tasic**	23,178	0.559	0.027	N/A	N/A	0	0.167	0.463
**Zyl (Human)**	24,023	0.108	**0.236**	N/A	**0.176**	**0.187**	**0.258**	0.106
**Zilionis (Human)**	34,558	0.708	0.416	N/A	0.698	0	0.219	**0.822**
**Wei**	41,565	0.476	0.321	N/A	**0.479**	**0.768**	0.305	**0.643**
**Cao**	90,579	0.247	**0.368**	N/A	N/A	**0.254**	N/A	**0.282**
**Orozco**	100,055	0.590	0	N/A	N/A	0.438	N/A	**0.763**
**Darrah**	162,490	0.337	0	N/A	N/A	N/A	N/A	**0.359**
**Mean ARI**		0.475	0.315	0.283	0.324	0.155	0.186	**0.499**

[1] N/A: Out of memory or error.

2.3 Assessment results using cluster analysis on real data without log transformation

We repeat the same process as the previous analysis, the only difference is we do not perform log transformation on the raw data before applying imputation method on it. The clustering results are also assessed using three different metrics Adjusted Rand Index (ARI), Jaccard Index (JI) and Purity Index (PI).

Figure S4A shows the ARI values obtained for data *without* log transformation. Again, the ARI values of scISR are consistently higher than those of raw data and of other methods in each grouping. Note that the ARI values of the raw data decrease (in comparison with ARI values obtained with log transformation). The reason is that the range of the data is very large. For example, the Deng dataset has a max RPKM value of 155,847 whereas the mean RPKM of the dataset is only 35. Without log transformation, genes with large values dominate the clustering analysis results, which is undesirable. A decrease in performance is observed for other imputation methods too (except scISR).

Table S5 shows the ARI values obtained for the raw data and the data inferred by the six imputation methods. In this analysis, scISR improves the clustering analysis in 24 out of 25 datasets by having the ARI values higher than those of the raw data. Among all methods, scISR has the highest average ARI values. Its average ARI value is 0.571, compare to 0.374, 0.356, 0.219, 0.307, 0.101 and 0.306 of the raw data, MAGIC's, scImpute's, SAVER's, scScope's, and scGNN's. A Wilcoxon test also confirms that the ARI values of scISR are significantly higher than those of raw data ($p = 6.3 \times 10^{-5}$) and of all other methods ($p = 1.9 \times 10^{-7}$).

Table S6 shows the JI values obtained for the raw data and the data inferred by the six imputation methods. In this analysis, scISR also improves the clustering analysis in 23 out of 25 datasets by having the JI values higher than those of the raw data. Among all methods, scISR has the highest average JI values. Its average JI value is 0.531, compare to 0.392, 0.399, 0.245, 0.308, 0.223, and 0.304 of the raw data, MAGIC's, scImpute's, SAVER's, scScope's, and scGNN's. A Wilcoxon test also confirms that the JI values of scISR are significantly higher than those of raw data ($p = 0.0001$) and of all other methods ($p = 4.4 \times 10^{-6}$).

Table S7 shows the PI values obtained from raw and imputed data. The results are similar to the analysis using ARI and JI. It is the only method that has the average PI value higher than that of the raw data. All other methods have an average PI less than that of the raw data. scISR improves cluster analysis by having PI values higher than those of the raw data in most datasets (21 out of 25). A Wilcoxon test also confirms that the PI values of scISR are significantly higher than those of raw data ($p = 0.0001$) and of all other methods ($p = 2.4 \times 10^{-7}$).

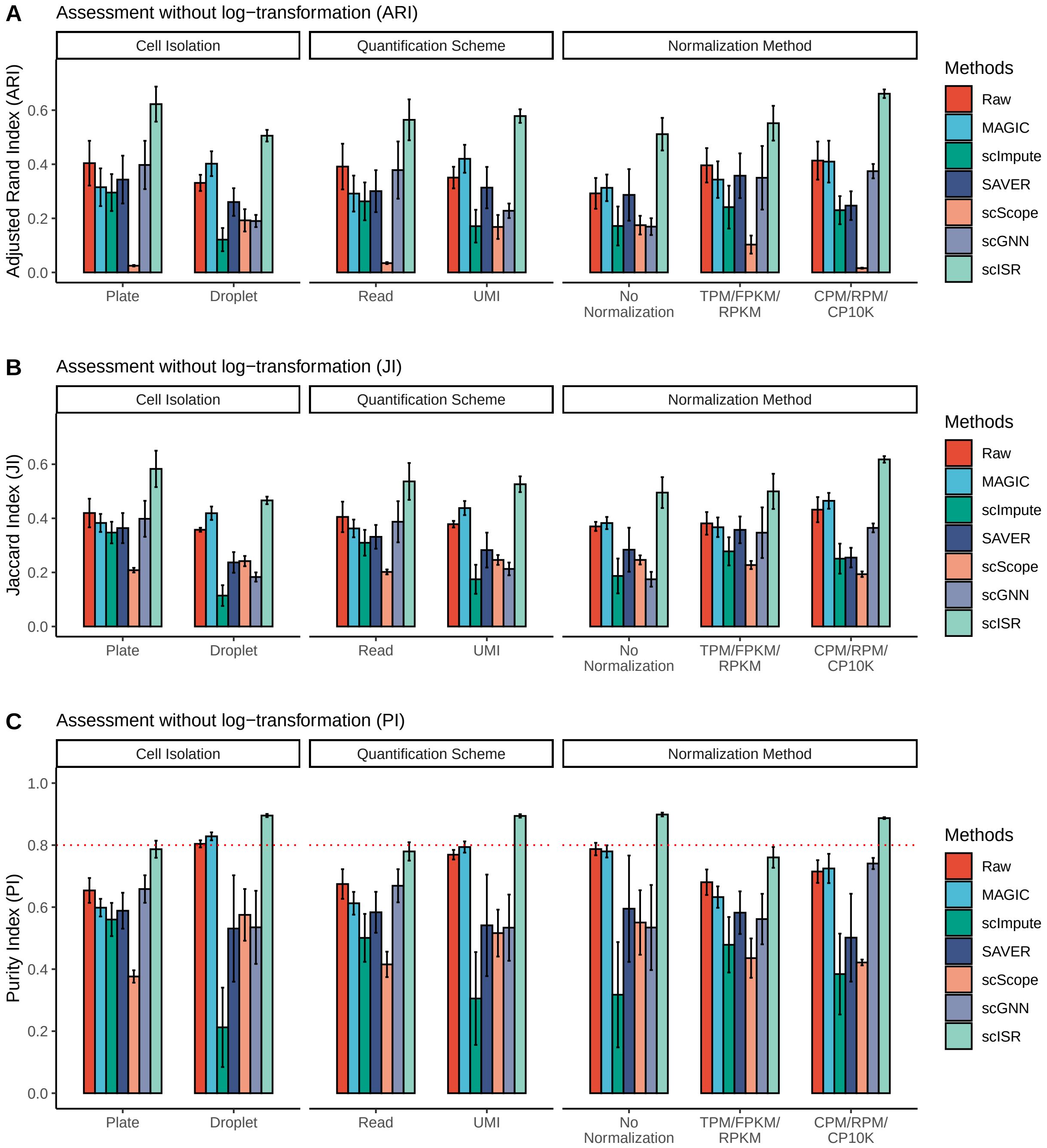

Figure S4. Assessment results of each imputation method with respect to cell isolation techniques, quantification schemes, or normalized units. The analysis is performed without a log transformation of the data. Panel (A) shows the results using Adjusted Rand Index (ARI) while panels (B) and (C) show the results using Jaccard Index (JI) and Purity Index (PI). scISR consistently outperforms other methods in every grouping by having the highest ARI, JI, and PI values.

Table S5. Adjusted Rand Index (ARI) obtained from raw and imputed data. In each row, a cell value is bold if the ARI value is higher than that of the raw data. scISR improves cluster analysis by having ARI values higher than those of the raw data in 24 out of 25 datasets. A Wilcoxon test also confirms that the ARI values of scISR are significantly higher than those of raw data ($p = 6.3 \times 10^{-5}$) and of all other methods ($p = 1.9 \times 10^{-7}$). The analysis is performed on data without log transformation.

Dataset	Size	Raw	MAGIC	scImpute	SAVER	scScope	scGNN	scISR
**Fan**	69	0.008	0	0	**0.015**	**0.017**	0.003	**0.249**
**Treutlein**	80	0.699	0.056	0	0	0.072	0.195	**0.758**
**Yan**	90	0.460	**0.705**	**0.547**	**0.609**	0.155	**0.884**	**0.768**
**Goolam**	124	0.629	0.17	0.281	0.379	0.112	**0.657**	**0.641**
**Deng**	268	0.359	0.263	**0.521**	**0.668**	0	**0.865**	**0.814**
**Pollen**	301	0.822	0.631	**0.826**	0.822	0.009	**0.833**	**0.955**
**Darmanis**	466	0.404	0.396	**0.458**	**0.472**	0	0.356	**0.705**
**Usoskin**	622	0.008	0.007	**0.353**	0.008	0.003	**0.127**	**0.87**
**Camp**	734	0.460	0.349	0.09	0.351	0.006	0.263	**0.462**
**Klein**	2,717	0.643	**0.66**	0.63	**0.852**	0.016	0.494	**0.984**
**Romanov**	2,881	0.193	**0.29**	**0.519**	**0.45**	0	**0.403**	**0.548**
**Segerstolpe**	3,514	0.079	**0.085**	**0.088**	**0.17**	0.003	**0.214**	**0.555**
**Manno**	4,029	0.167	**0.183**	**0.176**	**0.231**	0	0.107	**0.269**
**Marques**	5,053	0.100	**0.179**	**0.181**	**0.231**	0.001	**0.124**	**0.206**
**Baron**	8,569	0.276	0.271	**0.331**	**0.471**	0.008	**0.284**	**0.557**
**Sanderson**	12,648	0.155	0.125	N/A	0.122	0.119	0.064	**0.162**
**Slyper**	13,316	0.409	**0.509**	**0.484**	**0.484**	**0.438**	0.145	**0.496**
**Zilionis (Mouse)**	15,939	0.419	**0.528**	N/A	**0.42**	0	0.375	**0.675**
**Tasic**	24,023	0.818	0.74	N/A	N/A	0	0.442	0.477
**Zyl (Human)**	23,178	0.381	**0.39**	N/A	0.379	0.378	0.268	**0.424**
**Zilionis (Human)**	34,558	0.424	**0.737**	N/A	N/A	0	0.261	**0.71**
**Wei**	41,565	0.616	**0.776**	N/A	0.537	0.514	0.292	**0.617**
**Cao**	90,579	0.426	0.316	N/A	N/A	0.269	N/A	**0.43**
**Orozco**	100,055	0.390	0.376	N/A	N/A	**0.394**	N/A	**0.415**
**Darrah**	162,490	0.000	**0.105**	N/A	N/A	N/A	N/A	**0.528**
**Mean ARI**		0.374	0.356	0.219	0.307	0.101	0.306	**0.571**

[1] N/A: Out of memory or error.

Table S6. Jaccard Index (JI) obtained from raw and imputed data. In each row, a cell value is bold if the JI value is higher than that of the raw data. scISR improves cluster analysis by having JI values higher than those of the raw data in 23 out of 25 datasets. A Wilcoxon test also confirms that the JI values of scISR are significantly higher than those of raw data ($p = 0.0001$) and of all other methods ($p = 4.4 \times 10^{-6}$). The analysis is performed on data without log transformation.

Dataset	Size	Raw	MAGIC	scImpute	SAVER	scScope	scGNN	scISR
**Fan**	69	0.187	0.182	0.181	0.187	0.183	0.182	**0.261**
**Treutlein**	80	0.673	0.333	0.312	0.312	0.337	0.288	**0.727**
**Yan**	90	0.418	**0.627**	**0.47**	**0.529**	0.235	**0.831**	**0.695**
**Goolam**	124	0.634	0.403	0.434	0.401	0.355	0.621	**0.643**
**Deng**	268	0.387	**0.406**	**0.544**	**0.649**	0.278	**0.834**	**0.78**
**Pollen**	301	0.728	0.518	**0.733**	0.728	0.11	**0.74**	**0.924**
**Darmanis**	466	0.364	0.363	**0.409**	**0.404**	0.146	0.295	**0.606**
**Usoskin**	622	0.188	**0.28**	**0.429**	0.188	**0.279**	**0.25**	**0.828**
**Camp**	734	0.395	0.359	0.226	0.358	0.212	0.254	**0.398**
**Klein**	2,717	0.606	**0.622**	0.591	**0.813**	0.283	0.49	**0.977**
**Romanov**	2,881	0.268	**0.346**	**0.484**	**0.418**	0.246	**0.356**	**0.485**
**Segerstolpe**	3,514	0.243	**0.245**	**0.247**	0.192	0.227	0.185	**0.464**
**Manno**	4,029	0.108	**0.116**	**0.113**	**0.144**	0.03	0.069	**0.168**
**Marques**	5,053	0.134	**0.172**	**0.174**	**0.19**	0.107	0.116	**0.168**
**Baron**	8,569	0.259	0.254	**0.303**	**0.379**	0.199	0.223	**0.445**
**Sanderson**	12,648	0.243	0.219	N/A	0.22	0.217	0.133	**0.256**
**Slyper**	13,316	0.393	**0.493**	**0.47**	**0.471**	**0.435**	0.208	**0.478**
**Zilionis (Mouse)**	15,939	0.372	**0.46**	N/A	0.372	0.11	0.352	**0.61**
**Tasic**	24,023	0.809	0.735	N/A	N/A	0.134	0.421	0.52
**Zyl**	23,178	0.287	**0.299**	N/A	**0.291**	**0.288**	0.206	**0.323**
**Zilionis (Human)**	34,558	0.389	**0.666**	N/A	N/A	0.083	0.257	**0.633**
**Wei**	41,565	0.535	**0.715**	N/A	0.455	0.439	0.278	0.535
**Cao**	90,579	0.374	0.321	N/A	N/A	0.273	N/A	**0.379**
**Orozco**	100,055	0.370	0.355	N/A	N/A	0.37	N/A	**0.395**
**Darrah**	162,490	0.444	**0.479**	N/A	N/A	N/A	N/A	**0.589**
**Mean**		0.392	0.399	0.245	0.308	0.223	0.304	**0.531**

[1] N/A: Out of memory or error.

Table S7. Purity Index (PI) obtained from raw and imputed data. scISR improves cluster analysis by having PI values higher than those of the raw data in 21 out of 25 datasets. A Wilcoxon test also confirms that the PI values of scISR are significantly higher than those of raw data ($p = 0.0001$) and of all other methods ($p = 2.4 \times 10^{-7}$). The analysis is performed on data without log transformation.

Dataset	Size	Raw	MAGIC	scImpute	SAVER	scScope	scGNN	scISR
**Fan**	69	0.394	0.364	0.364	**0.409**	0.364	0.364	**0.545**
**Treutlein**	80	0.800	0.55	0.538	0.538	0.562	0.638	**0.838**
**Yan**	90	0.767	**0.8**	**0.822**	**0.8**	0.544	**0.911**	**0.844**
**Goolam**	124	0.823	0.613	0.702	0.782	0.565	**0.839**	0.823
**Deng**	268	0.713	0.608	**0.72**	**0.765**	0.504	**0.854**	**0.84**
**Pollen**	301	0.870	0.761	0.87	0.87	0.233	**0.884**	**0.963**
**Darmanis**	466	0.674	0.624	**0.697**	**0.721**	0.296	0.659	**0.848**
**Usoskin**	622	0.376	**0.383**	**0.595**	0.376	**0.378**	**0.518**	**0.929**
**Camp**	734	0.738	0.542	0.396	0.54	0.313	0.55	**0.74**
**Klein**	2,717	0.803	**0.81**	**0.81**	**0.883**	0.363	0.688	**0.991**
**Romanov**	2,881	0.578	**0.642**	**0.695**	**0.759**	0.354	**0.737**	**0.861**
**Segerstolpe**	3,514	0.518	**0.531**	**0.519**	**0.685**	0.376	**0.713**	**0.847**
**Manno**	4,029	0.394	**0.407**	0.381	**0.416**	0.102	0.296	**0.506**
**Marques**	5,053	0.353	**0.461**	**0.427**	**0.453**	0.185	**0.37**	**0.445**
**Baron**	8,569	0.752	0.741	0.747	**0.863**	0.302	0.749	**0.947**
**Sanderson**	12,648	0.936	0.927	N/A	0.914	0.869	0.879	**0.958**
**Slyper**	13,316	0.907	0.903	0.894	0.899	0.85	0.706	**0.917**
**Zilionis (Mouse)**	15,939	0.873	**0.971**	N/A	0.873	0.503	0.797	**0.973**
**Tasic**	24,023	0.931	0.922	N/A	N/A	0.485	**0.934**	0.856
**Zyl**	23,178	0.861	0.854	N/A	0.784	0.8	0.754	**0.875**
**Zilionis (Human)**	34,558	0.749	**0.918**	N/A	N/A	0.37	0.701	**0.92**
**Wei**	41,565	0.768	**0.772**	N/A	0.75	0.743	0.561	0.768
**Cao**	90,579	0.776	0.669	N/A	N/A	0.595	0	0.761
**Orozco**	100,055	0.935	**0.951**	N/A	N/A	**0.94**	0	**0.928**
**Darrah**	162,490	0.710	**0.764**	N/A	N/A	N/A	0	**0.942**
**Mean**		0.720	0.700	0.407	0.563	0.464	0.604	**0.835**

[1] N/A: Out of memory or error.

2.4 Absolute correlation between good genes and imputable genes

Figure S5 shows the distribution of absolute correlations between the imputable and good genes. The horizontal axis shows the number of genes used for calculating the absolute correlation while the vertical axis shows the absolute correlation. Each box represents the absolute correlation values obtained across all of the 25 datasets. With 10 genes selected, the median correlation is 0.75 and this value drops to 0.25 when we select the first quartile.

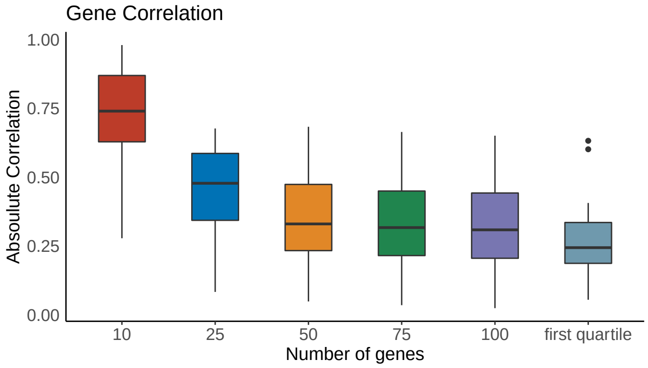

Figure S5. Distribution of absolute correlations between the imputable and good genes. The horizontal axis shows the number of genes while the vertical axis shows the absolute correlation. Each box represents the absolute correlation values obtained across all of the 25 datasets. With 10 genes selected, the median correlation is 0.75 and this value drops to 0.25 when we select the first quartile.

3 Transcriptome landscape visualization

To visualize the transcriptome landscapes, we plotted the transcriptome landscapes obtained from the raw and the imputed data. The landscapes generated by t-SNE are shown in Figures S6–S10 while those generated by UMAP are shown in Figures S11–S15. For each dataset, we plotted seven panels (from left to right): one for the raw data and six for the imputed data obtained from MAGIC, scImpute, SAVER, scScope, scGNN, and scISR.

In principle, the imputation should not significantly alter the original transcriptome landscape. In order to assess how similar the landscape of the imputed data to the original landscape, we calculated the distance correlation[15] between the new landscapes and the original landscape. The higher the distance correlation, the more similar the new landscape is to the original landscape. We show this value on top of each imputed landscape.

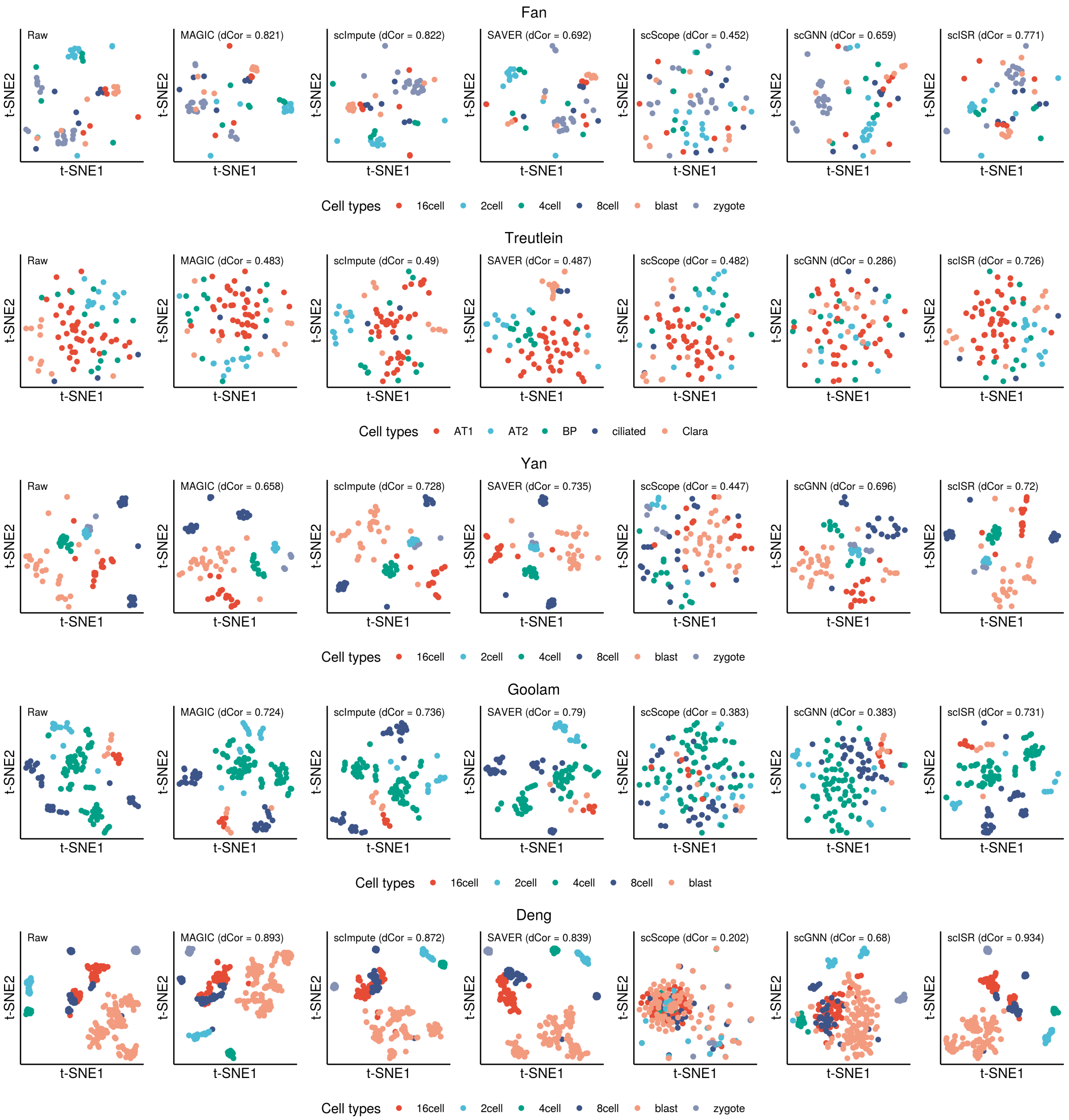

Figure S6. Transcriptome landscape of the Fan, Treutlein, Yan, Goolam and Deng datasets (top to bottom) using t-SNE. Different colors code for different cell types. The distance correlation calculated for each imputed dataset shows the similarity between the new landscape (from imputed data) and the original landscape (from raw data).

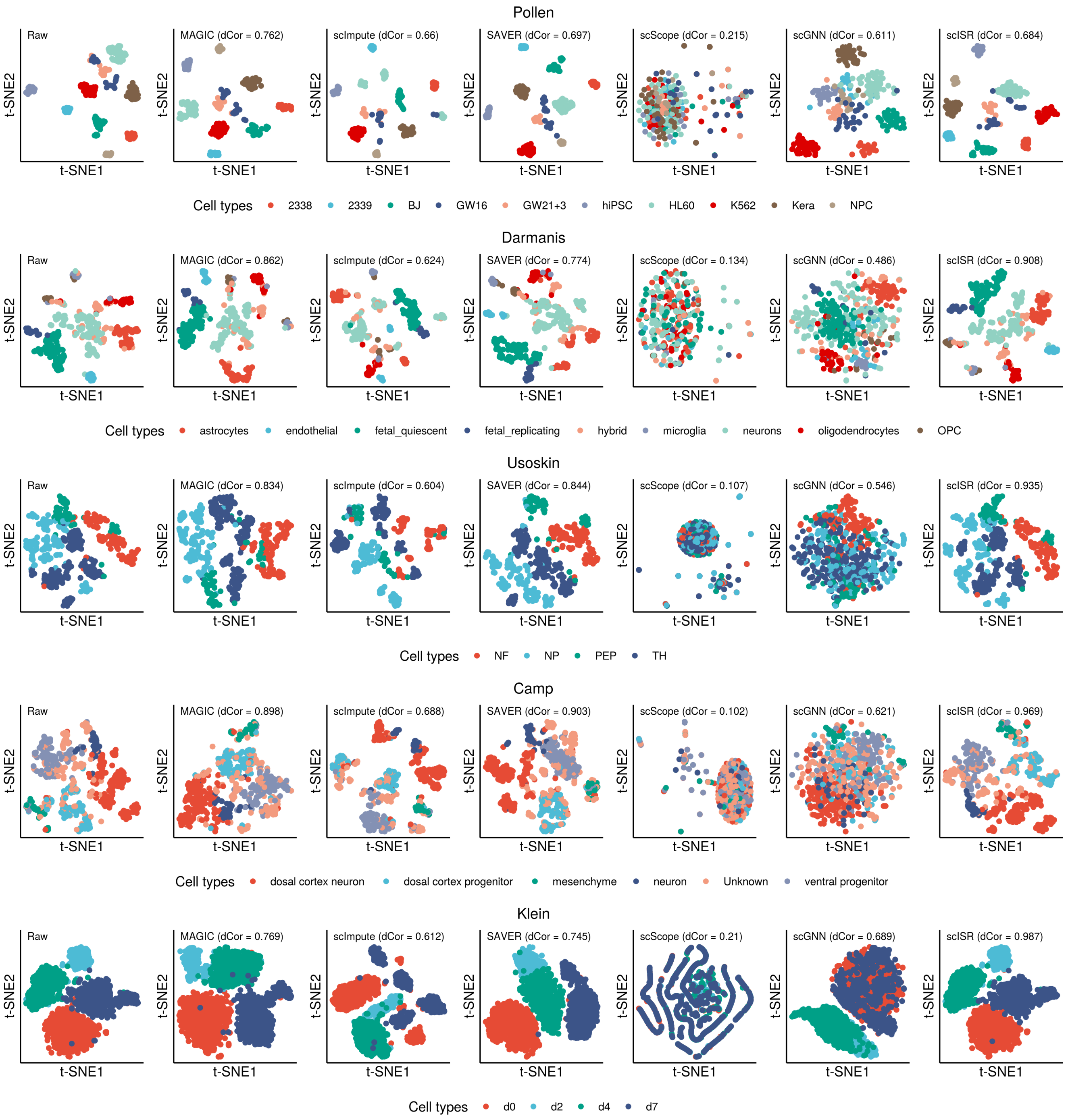

Figure S7. Transcriptome landscape for the Pollen, Darmanis, Usoskin, Camp and Klein datasets (top to bottom) using t-SNE. Different colors code for different cell types. The distance correlation calculated for each imputed dataset shows the similarity between the new landscape (from imputed data) and the original landscape (from raw data).

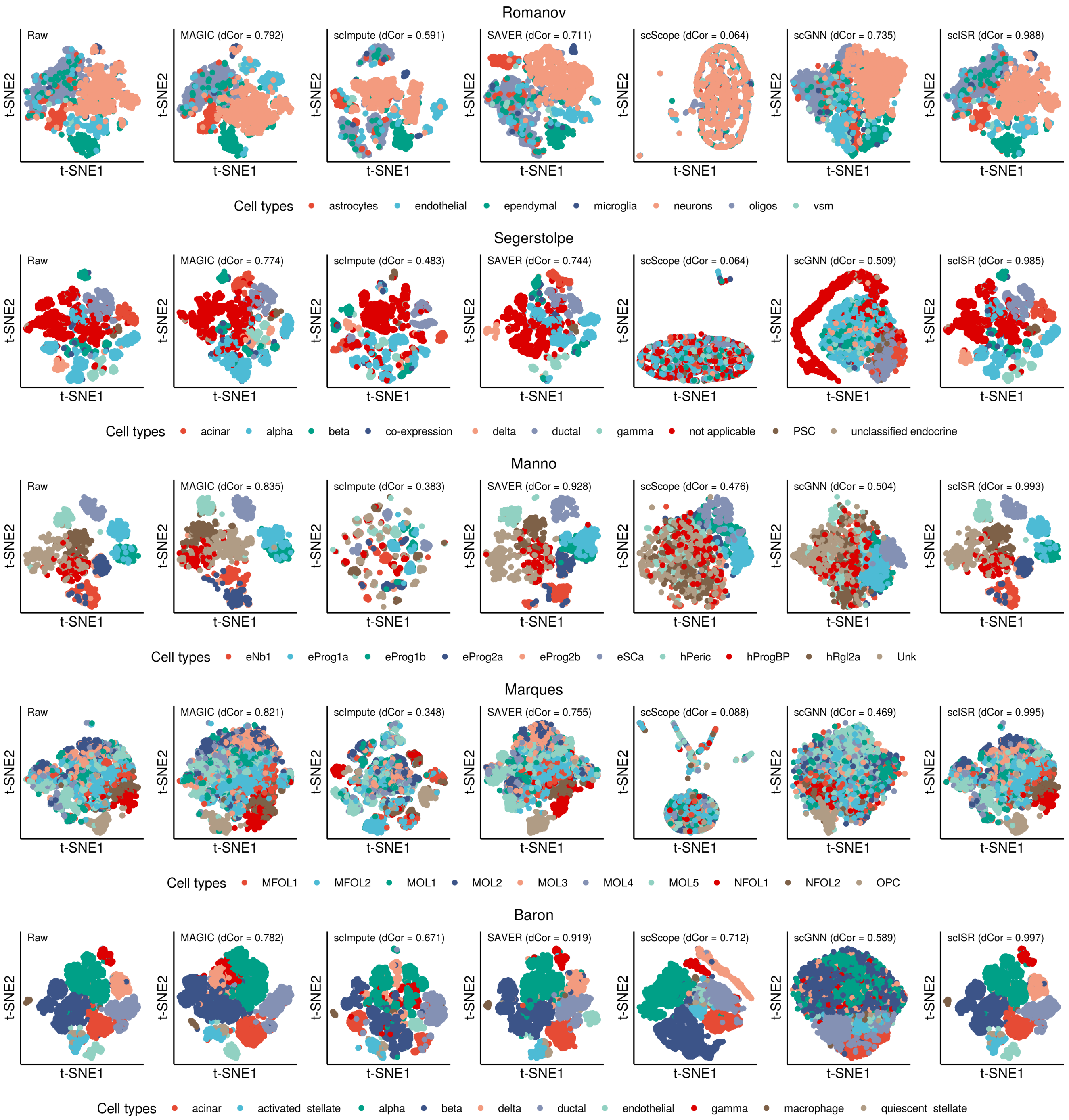

Figure S8. Transcriptome landscape for the Romanov, Segerstolpe, Manno (Human), Marques and Barron (Human) datasets (top to bottom) using t-SNE. Different colors code for different cell types. The distance correlation calculated for each imputed dataset shows the similarity between the new landscape (from imputed data) and the original landscape (from raw data).

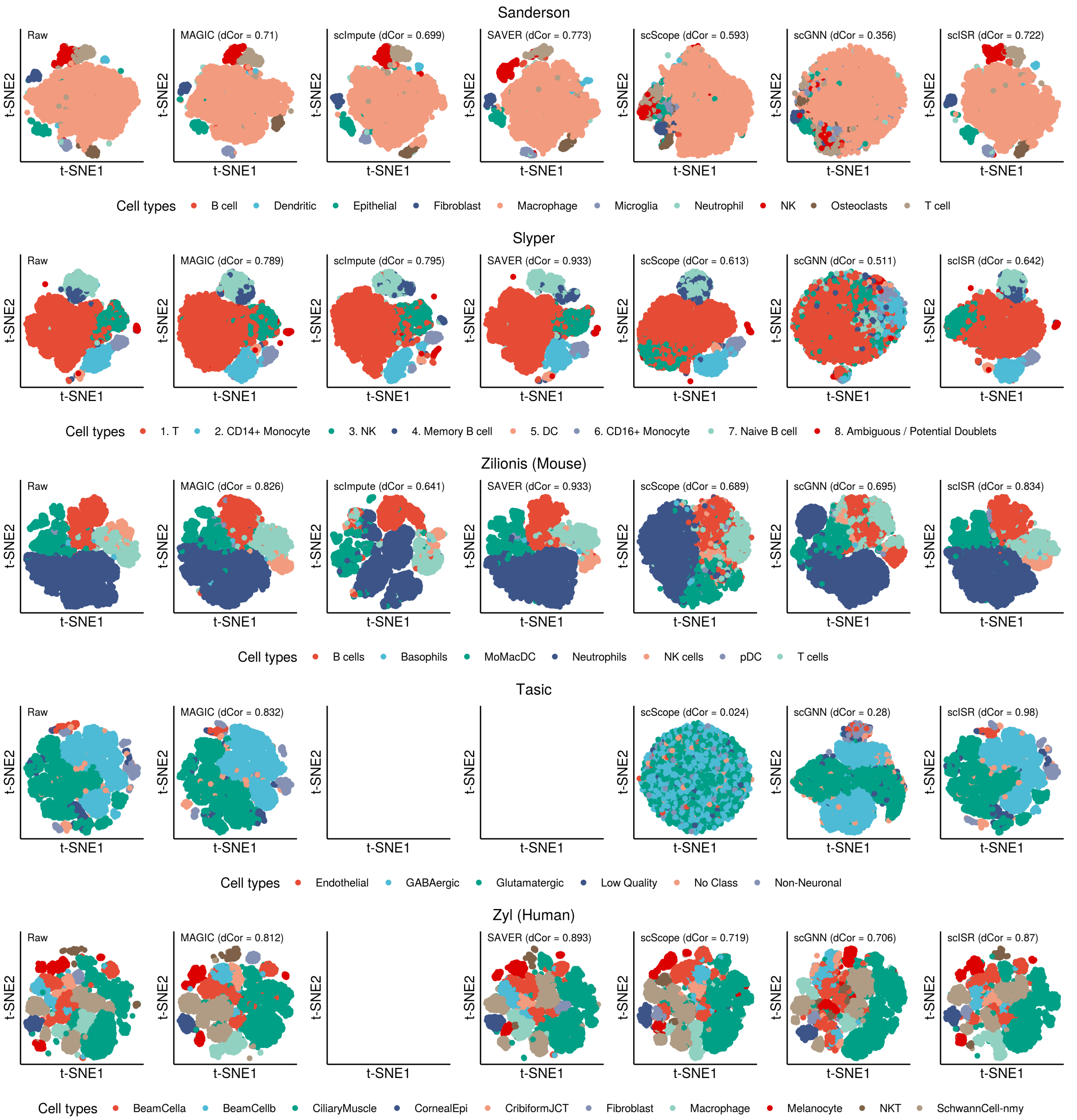

Figure S9. Transcriptome landscape for the Sanderson, Slyper, Zilionis (Mouse), Tasic and Zyl (Human) datasets (top to bottom) using t-SNE. Different colors code for different cell types. The distance correlation calculated for each imputed dataset shows the similarity between the new landscape (from imputed data) and the original landscape (from raw data).

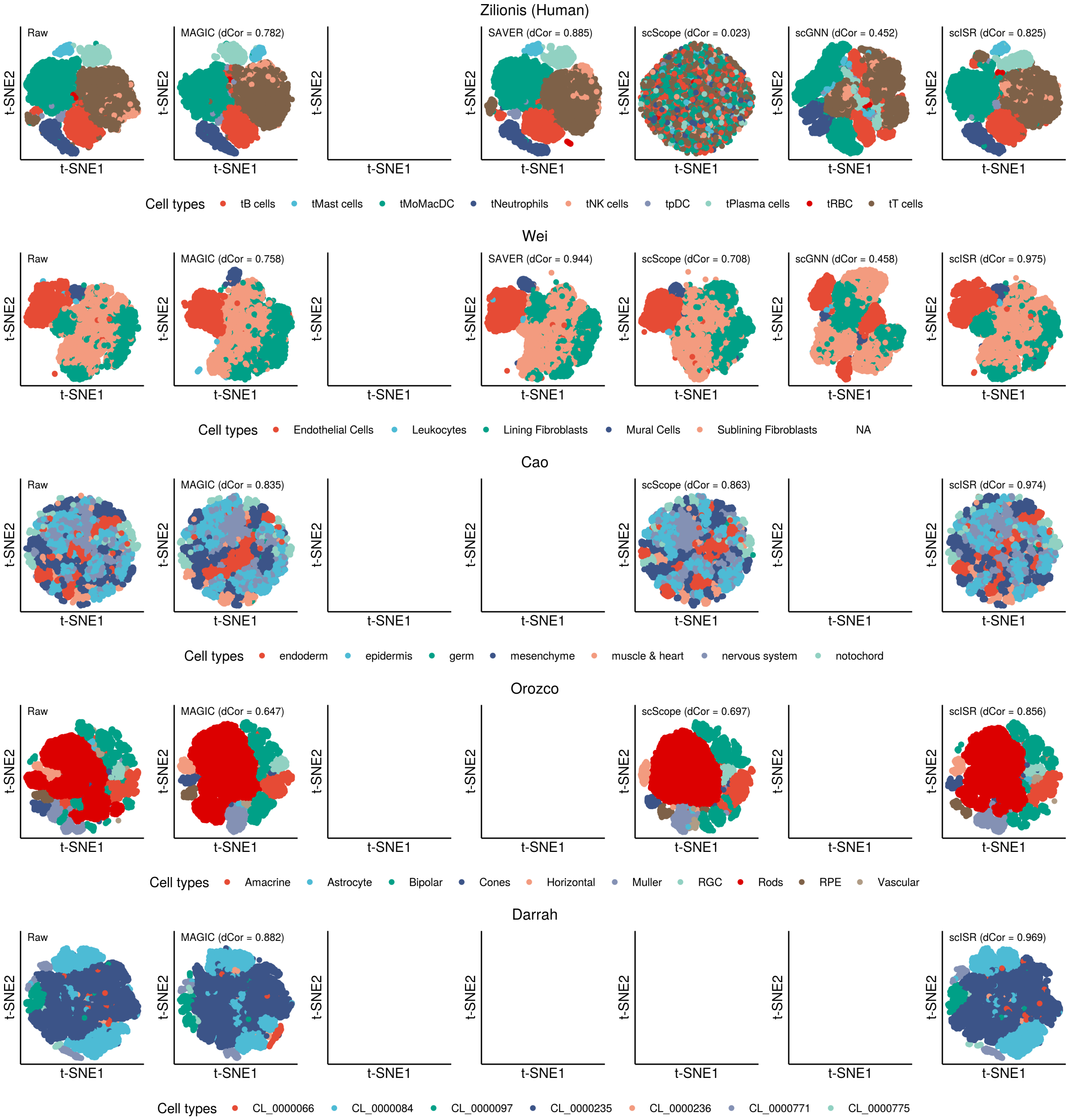

Figure S10. Transcriptome landscape for the Zillionis (Human), Wei (Human), Cao, Orozco and Darrah datasets (top to bottom) using t-SNE. Different colors code for different cell types. The distance correlation calculated for each imputed dataset shows the similarity between the new landscape (from imputed data) and the original landscape (from raw data).

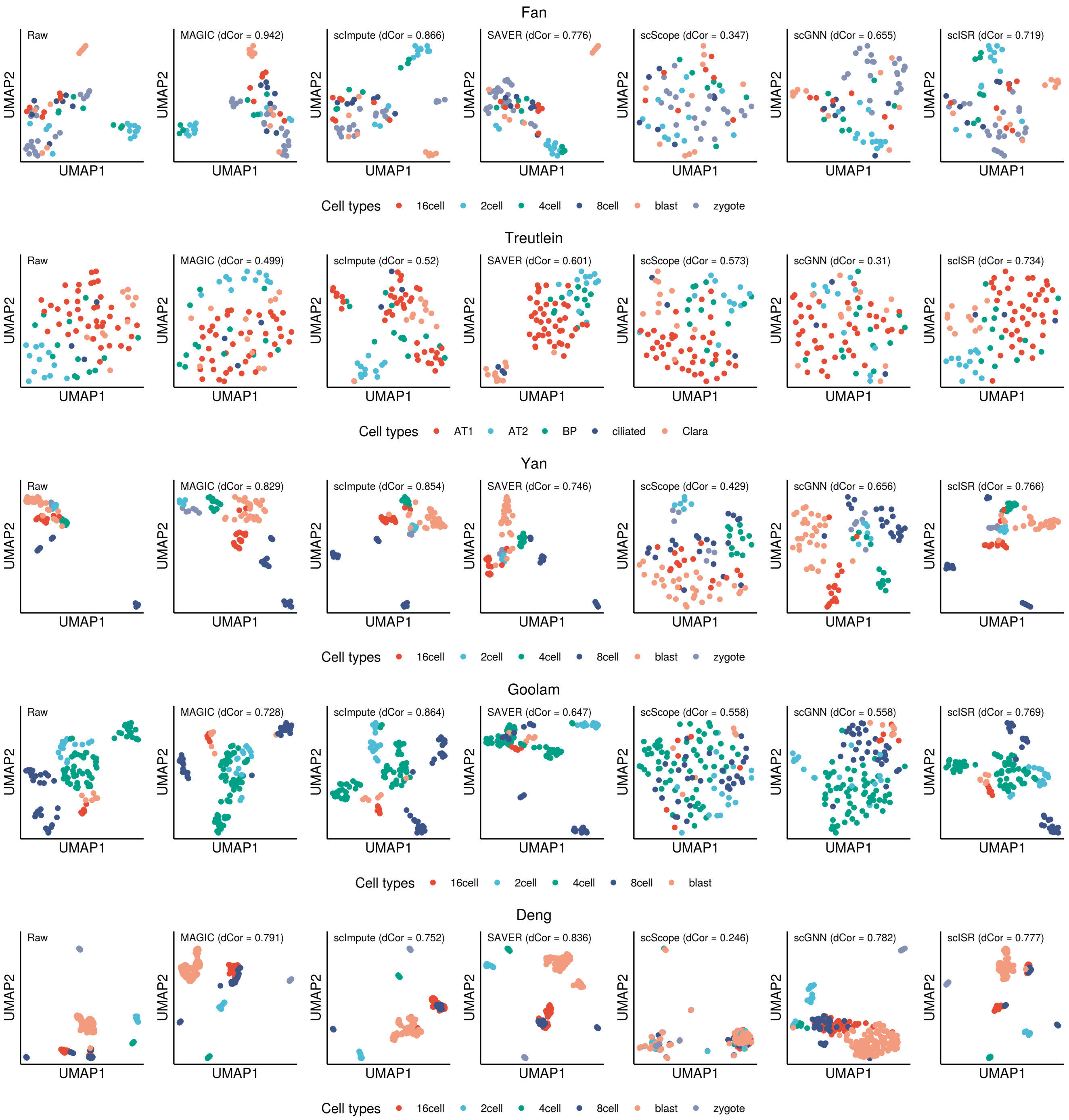

Figure S11. Transcriptome landscape for the Fan, Treutlein, Yan, Goolam and Deng datasets (top to bottom) using UMAP. Different colors code for different cell types. The distance correlation calculated for each imputed dataset shows the similarity between the new landscape (from imputed data) and the original landscape (from raw data).

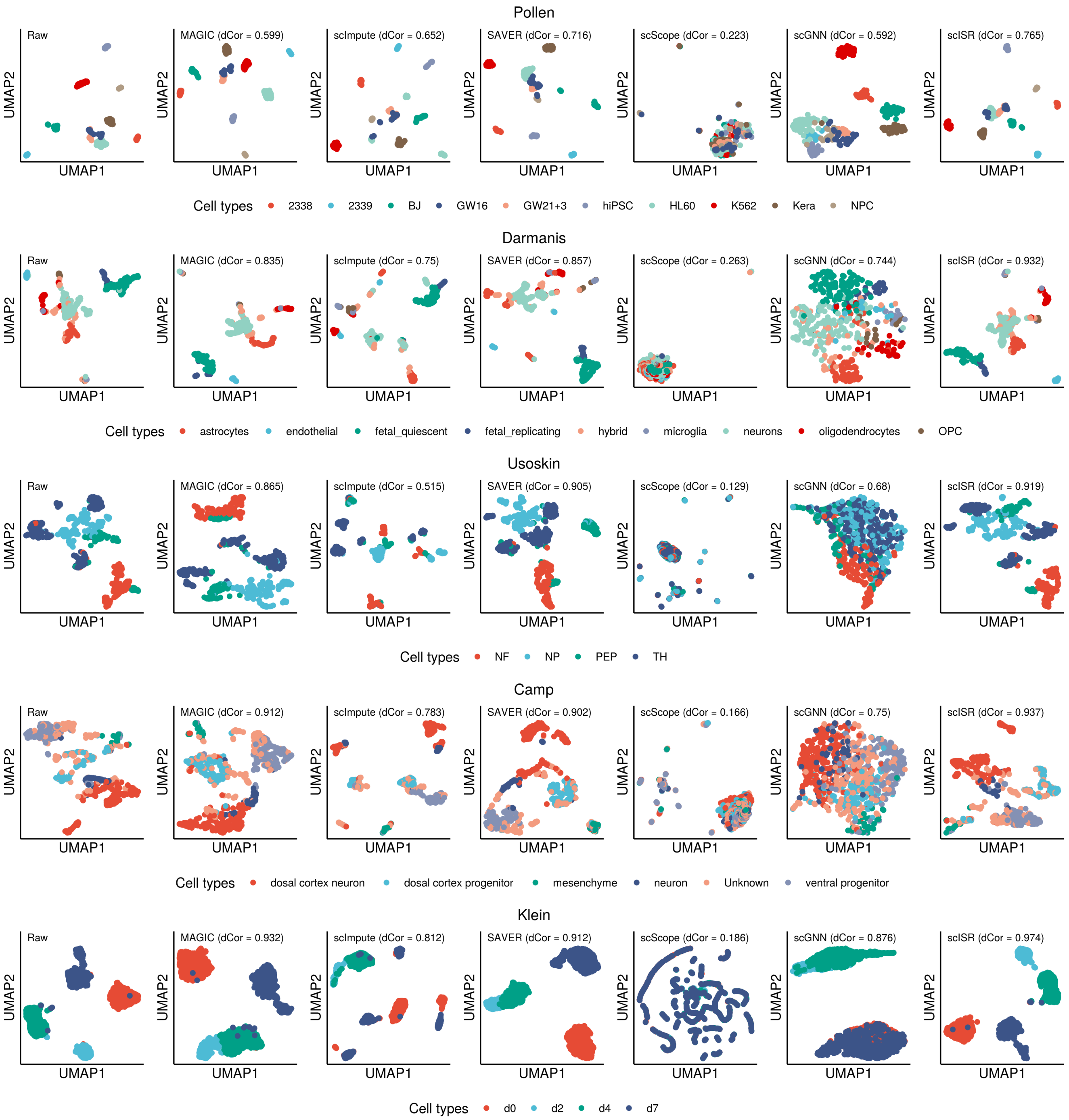

Figure S12. Transcriptome landscape for the Pollen, Darmanis, Usoskin, Camp and Klein datasets (top to bottom) using UMAP. Different colors code for different cell types. The distance correlation calculated for each imputed dataset shows the similarity between the new landscape (from imputed data) and the original landscape (from raw data).

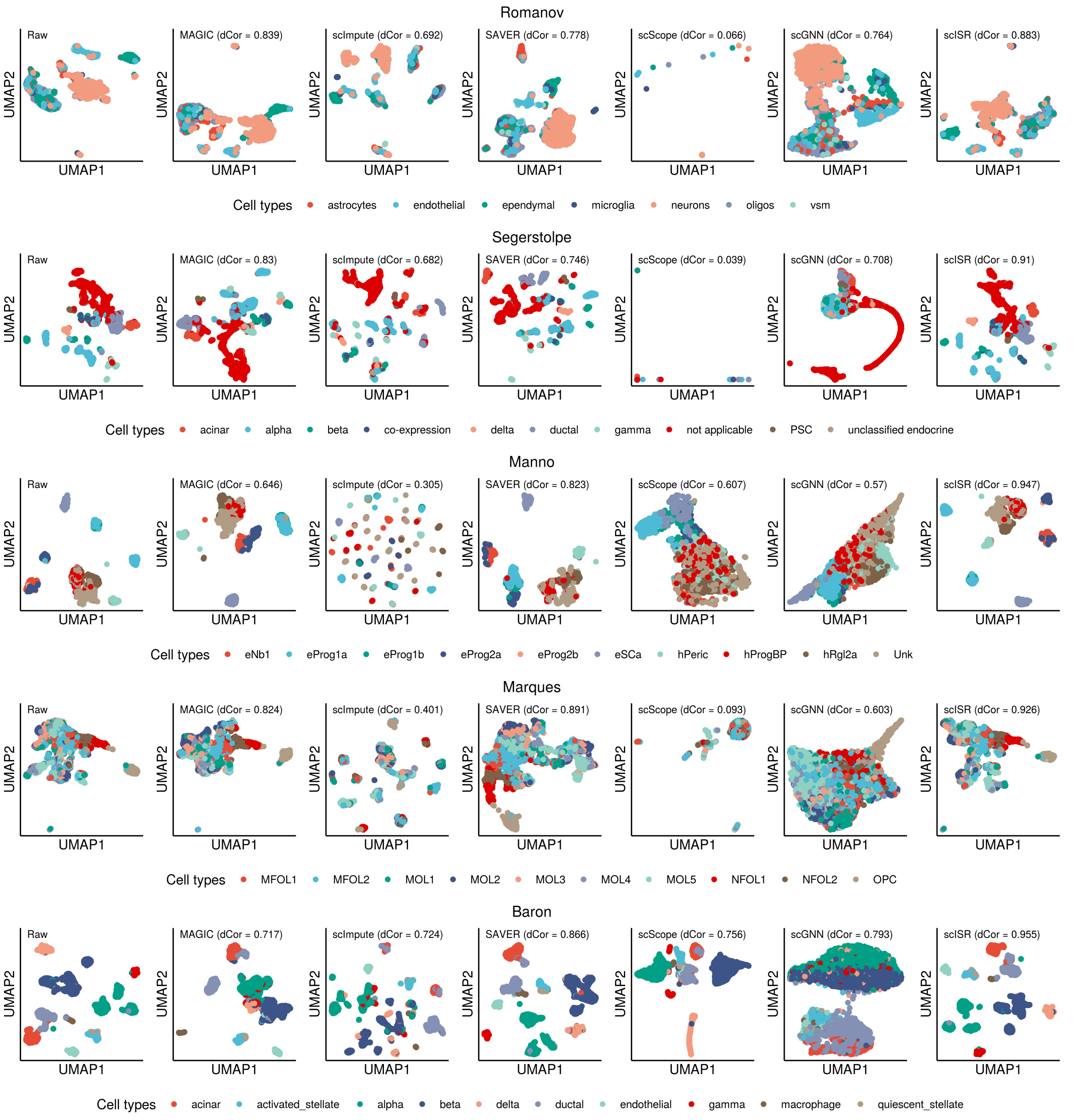

Figure S13. Transcriptome landscape for the Romanov, Segerstolpe, Manno (Human), Marques and Barron (Human) datasets (top to bottom) using UMAP. Different colors code for different cell types. The distance correlation calculated for each imputed dataset shows the similarity between the new landscape (from imputed data) and the original landscape (from raw data).

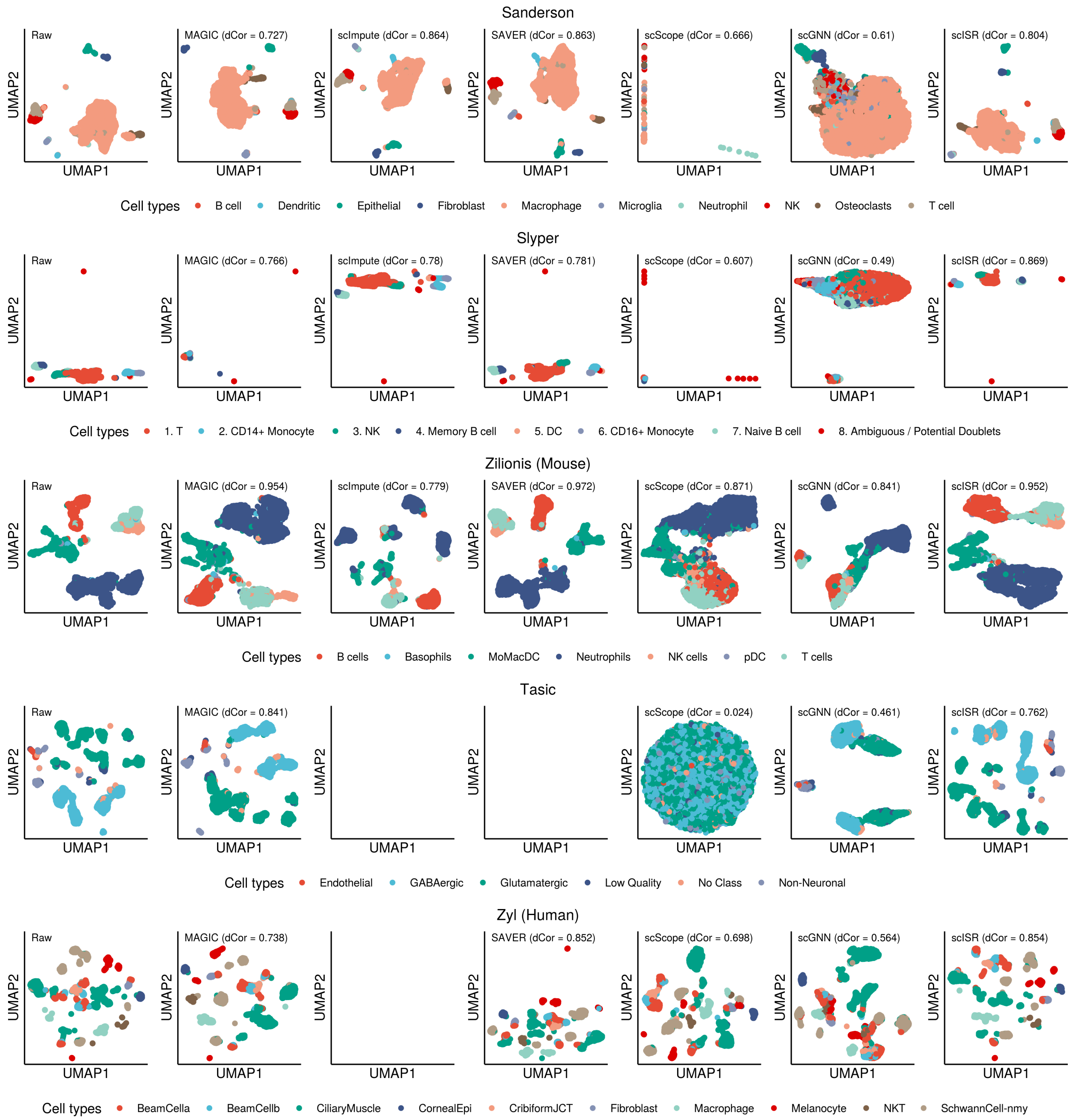

Figure S14. Transcriptome landscape for the Sanderson, Slyper, Zilionis (Mouse), Tasic and Zyl (Human) datasets (top to bottom) using UMAP. Different colors code for different cell types. The distance correlation calculated for each imputed dataset shows the similarity between the new landscape (from imputed data) and the original landscape (from raw data).

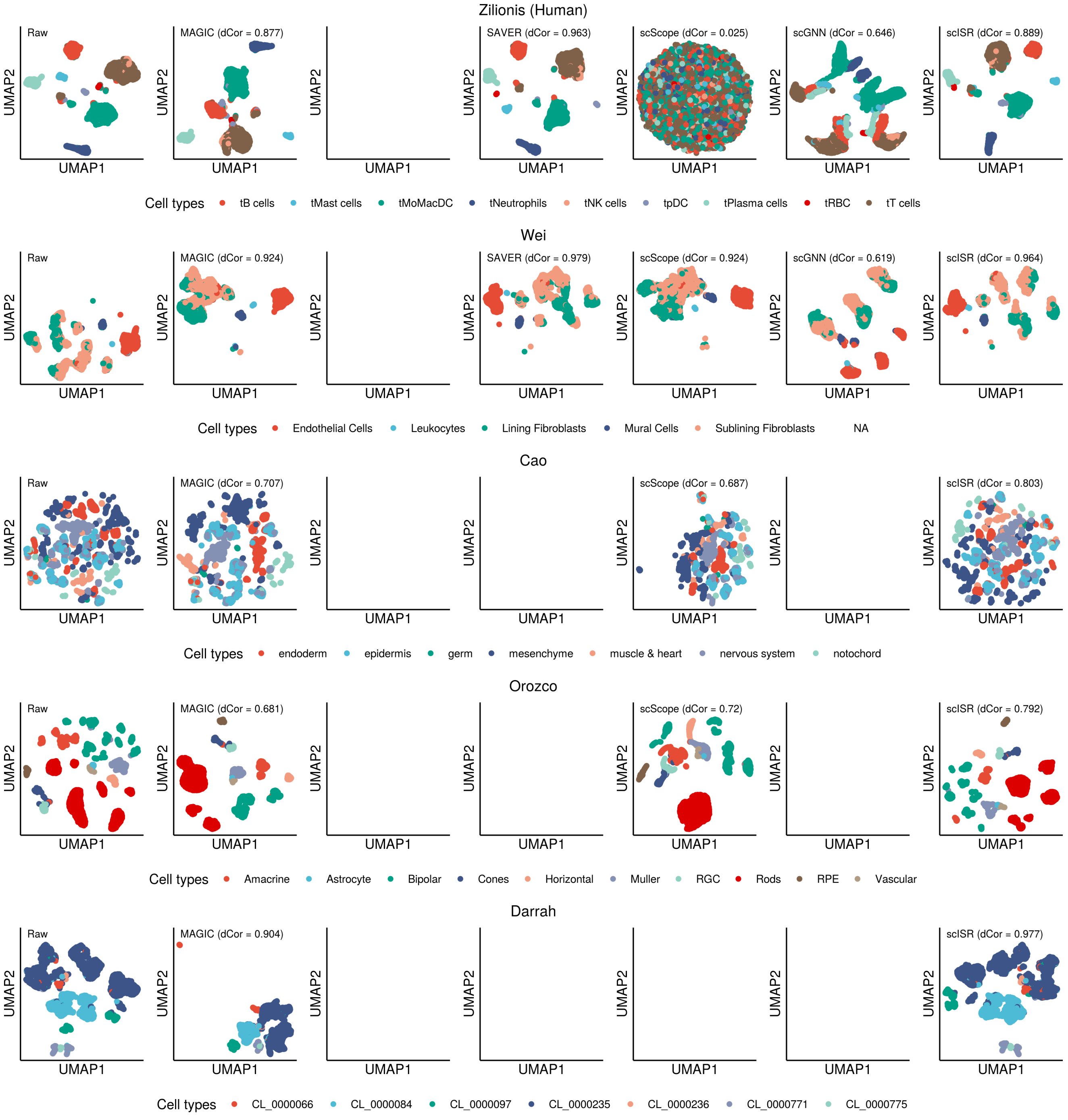

Figure S15. Transcriptome landscape for the Zillionis (Human), Wei (Human), Cao, Orozco and Darrah datasets (top to bottom) using UMAP. Different colors code for different cell types. The distance correlation calculated for each imputed dataset shows the similarity between the new landscape (from imputed data) and the original landscape (from raw data).

4 Simulation studies

4.1 Case studies

To present a comprehensive simulation analysis, we generate a number of simulations by varying the number of cells from 100 to 10,000 and the number of genes from 300 to 10,000. The cells/genes combination setups are presented as follows: 100×300, 1,000×3,000, 3,000×9,000, 5,000×10,000, 7,000×10,000, and 10,000×10,000.

In each of the 6 datasets, the expression values follow a normal distribution $\mathcal{N}(\mu, \sigma)$. We set $\mu = 1$ and $\sigma = 0.15$. We slightly shift the mean of the cells and genes to create 4 different cell types and 3 gene groups – each cell type has an equal number of cells. We name this data as *complete data* and use the expression values as the ground truth for benchmarking. Next, we introduce the dropout events. We randomly select 40% of the genes and consider those as genes that are impacted by dropout events. We randomly assign 30% of the values of these genes to zero. We name this data as *masked data*.

In these case studies, we present a detailed simulation results for 3 datasets: 100×300, 1,000×3,000 and 10,000×10,000. Panels A and B in Figures S16, S17 and S18 show the simulation data for the setting of 100×300, 1,000×3,000 and 10,000×10,000, respectively. In each figure, panel A shows the transcriptome landscape of the complete data and panel B shows the masked data. In each dataset, the transcriptome landscape and gene-cell heatmap of the *complete data* clearly show the presence of three cell types and four gene groups. With *masked data*, dropout events clearly alter the cells' transcriptome landscape, making it difficult to separate the cell types. The ultimate goal of imputation is to infer the masked (dropout) values in order to recover the original transcriptome landscape and expression profile.

We apply the six imputation methods on the *masked data* and assess the quality of the imputed data by comparing them against the ground truth. Panels C, D, E, F, G, and H in Figures S16, S17 and S18 show the data imputed by MAGIC, scImpute, SAVER, scScope, scGNN, and scISR, respectively.

MAGIC imputes the missing values by smoothing the expression values. The heatmap clearly shows that many expression values, including non-zero-valued entries, were altered by MAGIC. Further, MAGIC drastically alters the transcriptome landscape, making the landscape of the imputed data very different from those of both *complete* and *masked data*. scImpute improves the quality of the data but is still not able to separate certain cell types, e.g., *Type 2* (yellow) and *Type 4* (green) in Supplementary Figure S16D. Also, the red color in scImpute's heatmap becomes lighter. This means that scImpute alters the values of non-zero entries to make the data better fit into the assumed mixture model. SAVER further improves the transcriptome landscape and separates the four cell types. Even though the yellow and green cells are close to one another, they can be completely separated. However, the heatmap color in SAVER's data does not completely match that of the *complete data*. There are still many blue stripes (dropout values) that remain uncorrected. Furthermore, there are many white stripes that do not exist in the original data, i.e., many dropout entries assume wrong values. scScope and scGNN oversmooth the imputed data such that it merges all the cells in four types together. The heatmaps clearly show that many expression values, including non-zero-valued entries, were altered by scScope and scGNN. In contrast, scISR is able to recover the transcriptome landscape as well as most of the missing values. The color patterns in the imputed data's heatmap are almost identical to the patterns in the *complete data*. scISR did not alter any non-zero entry and recovered most of the dropout values. The transcriptome landscapes of scISR-imputed data (panels H) are similar to those of the complete data (panels A).

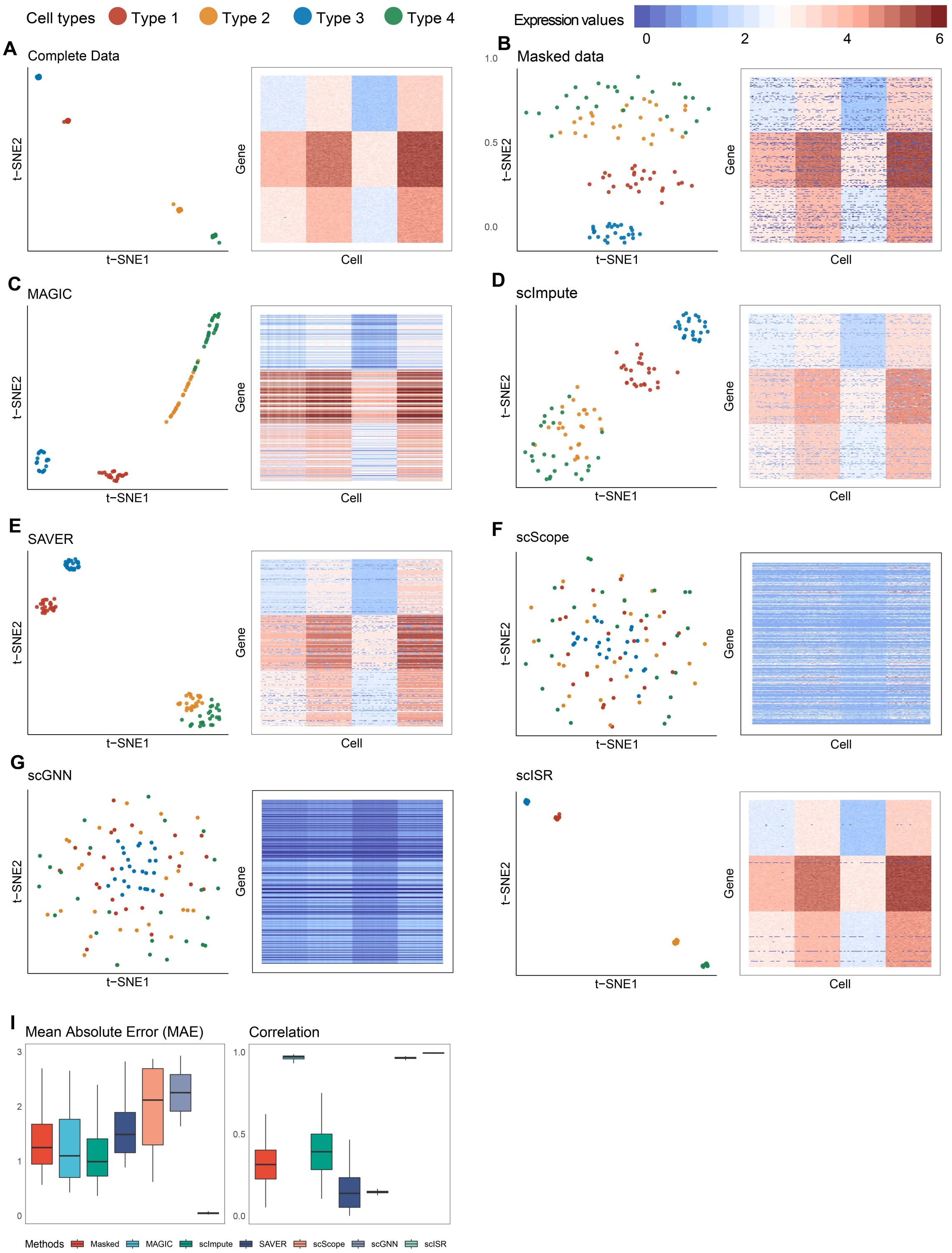

Figure S16. Assessment of MAGIC, scImpute, SAVER, and scISR using simulation (100 cells and 300 genes). (A) – (H) The visualization of the *complete data*, *masked data* and *imputed data* recovered by MAGIC, scImpute, SAVER, scScope, scGNN, and scISR. In each subfigure, the left panel shows the transcriptome landscape using t-SNE while the right panel shows the gene-cell heatmap. (I) Mean Absolute Error (MAE) and correlation coefficients obtained by comparing masked/imputed data with the complete data. We calculate the MAE and correlation values for each gene and then plot the distributions of each metric using boxplot. The transcriptome landscapes and heatmaps show that scISR comes closest to recovering the complete data. scISR also has significantly smaller MAE values as well as significantly higher correlation coefficients than other methods with p-values 1.6×10^{-64} and 9.2×10^{-63}, respectively (Wilcoxon test).

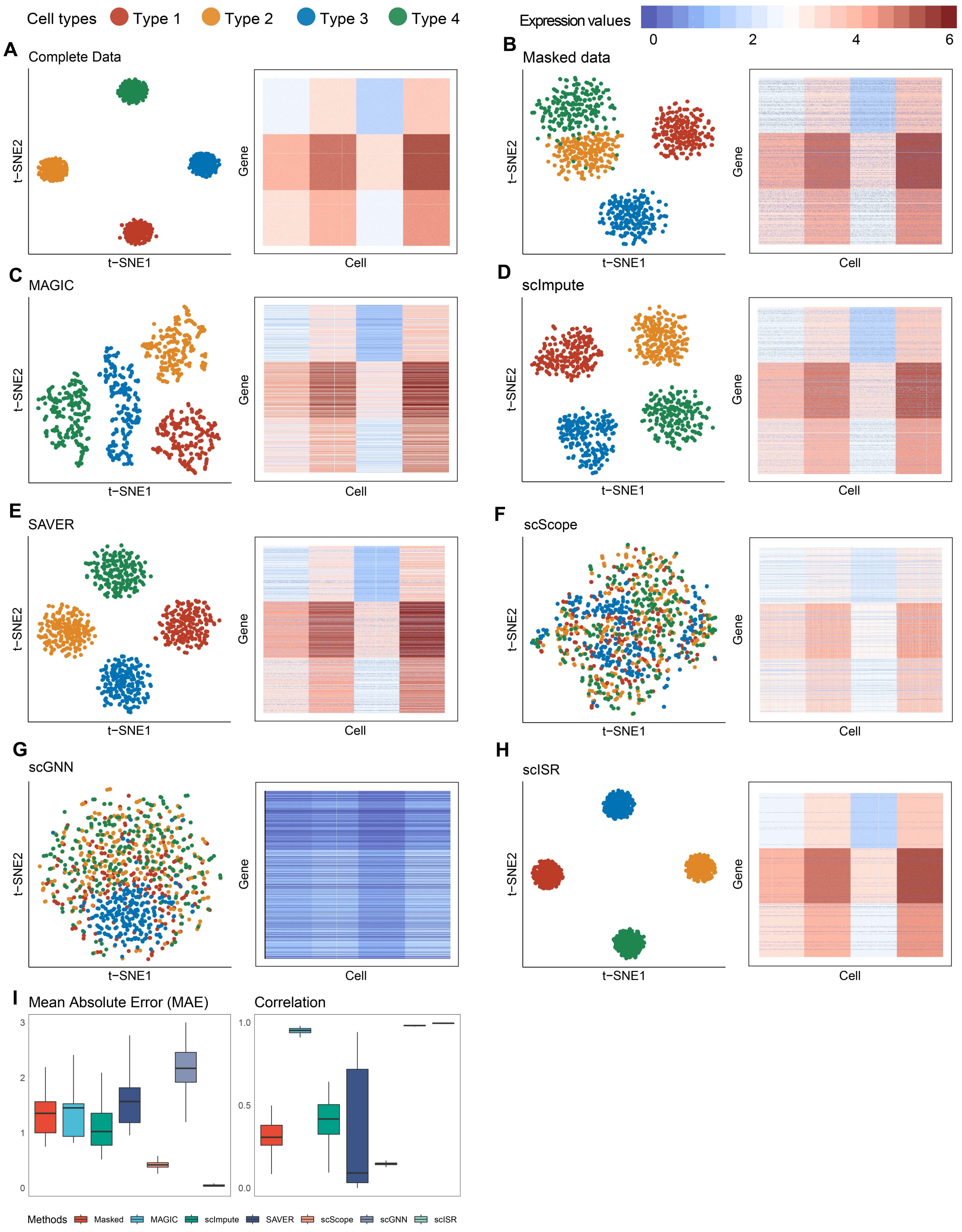

Figure S17. Assessment of MAGIC, scImpute, SAVER, and scISR using simulation of 1,000 cells. (A) – (H) The visualization of the *complete data*, *masked data* and *imputed data* recovered by MAGIC, scImpute, SAVER, scScope, scGNN, and scISR. In each subfigure, the left panel shows the transcriptome landscape using t-SNE while the right panel shows the gene-cell heatmap. (I) Mean Absolute Error (MAE) and correlation coefficients obtained by comparing masked/imputed data with the complete data. We calculate the MAE and correlation values for each gene and then plot the distributions of each metric using boxplot. The transcriptome landscapes and heatmaps show that scISR comes closest to recovering the complete data. scISR also has significantly smaller MAE values as well as significantly higher correlation coefficients than other methods with p-values $< 10^{-100}$ and $< 10^{-100}$, respectively (using Wilcoxon test).

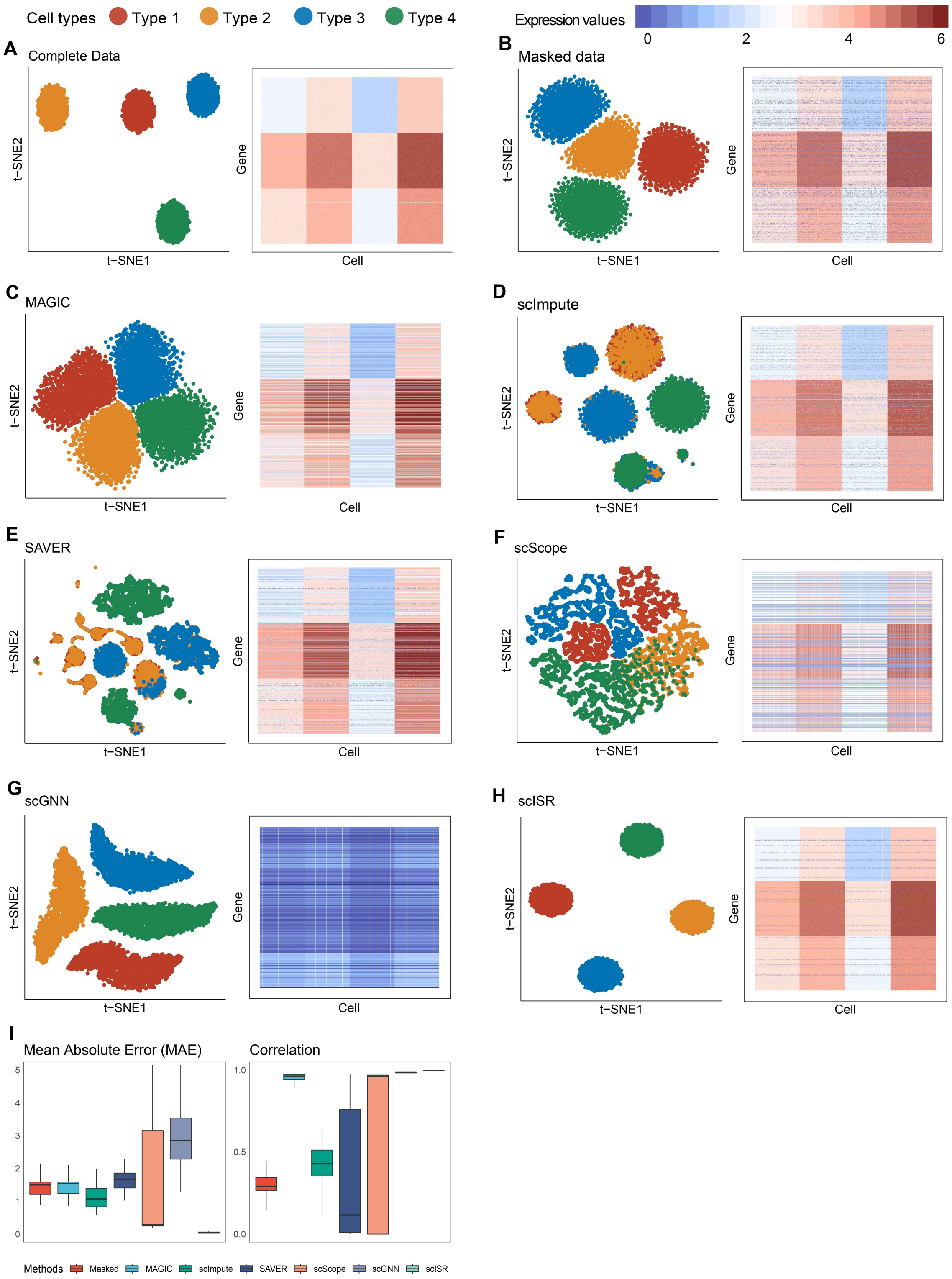

Figure S18. Assessment of MAGIC, scImpute, SAVER, and scISR using simulation of 10,000 cells. (A) – (H) The visualization of the *complete data*, *masked data* and *imputed data* recovered by MAGIC, scImpute, SAVER, scScope, scGNN, and scISR. In each subfigure, the left panel shows the transcriptome landscape using t-SNE while the right panel shows the gene-cell heatmap. (I) Mean Absolute Error (MAE) and correlation coefficients obtained by comparing masked/imputed data with the complete data. We calculate the MAE and correlation values for each gene and then plot the distributions of each metric using boxplot. The transcriptome landscapes and heatmaps show that scISR comes closest to recovering the complete data. scISR also has significantly smaller MAE values as well as significantly higher correlation coefficients than other methods with p-values $< 10^{-100}$ and $< 10^{-100}$, respectively (using Wilcoxon test).

4.2 Robustness of scISR against non-uniform dropout probability

To further investigate the robustness of the hypergeometric test embedded in scISR, we have also performed additional simulation studies with different sample sizes and dropout scenarios. In these simulations, we know the ground truth and the underlying probability distributions. Therefore, we can properly assess the reliability of scISR when the dropout probability is not uniformly distributed.

First, we generate a new dataset that consists of 1,000 samples and 3,000 genes – all expression values follow a normal distribution $N(\mu, \sigma)$ where $\mu = 1$ and $\sigma = 0.15$. We slightly shift the mean of the cells and genes to create 4 different cell types. We name this as *complete data*. Next, we randomly assign dropout values to the data in two different scenarios. In the first scenario, the dropout probability is uniformly distributed. In the second scenario, the dropout probability follows a normal distribution. For example, at 60% dropout rate, the dropout probability follows a distribution of $N(0.6, 0.1)$. To make the simulation more general, we vary the number of cells (from 1,000 to 10,000), the number of genes (from 3,000 to 10,000), and the dropout rate (from 60% to 90%). We name the data with dropouts as *masked data*. Next, we impute the *masked data* using six imputation methods to obtain the *imputed data*. Finally, to assess the performance of imputation methods, we compare the imputed data against the complete data using Mean Absolute Error (MAE) and correlation coefficients.

The top left panel in Figure S19 shows the MAE values obtained for datasets with 1,000 cells and 3,000 genes. In this panel, the left side displays the results obtained for uniform distributions while the right side shows the results for the normal distributions. When the dropout probability is uniformly distributed, scISR is able to recover most of the dropout values, resulting in a median MAE close to zero at any dropout rate. When the dropout probability is normally distributed, scISR still performs as well at 60% to 80% dropout but it becomes less accurate at 90% rate. At 90% dropout rate, scISR recovers only a part of the data (median MAE of approximately 2.11 compared to 3.65 of masked data). Assessment results using correlation coefficient (top right panel) also confirm our finding. However, as seen in Figure S19, the result of scISR is still much better than other imputation methods.

The next two panels (second row) in Figure S19 show the results obtained for datasets with 3,000 cells and 9,000 genes. scISR is more accurate (lower MAE and higher correlation) for these datasets compared to datasets with 1,000 cells. At dropout rates of 60%, 70%, and 80%, scISR performs consistently well for uniform and normal distributions alike (median MAE value close to zero). At 90% rate, the median MAE of scISR for normal distributions is now 1.61 (compared to 2.11 for datasets with 1,000 cells and 3,000 genes). The reason for such improvement is that with the same dropout rate, larger datasets provide us with more data to learn from, leading to improved hypothesis testing (hypergeometric test) and prediction (linear regression). For datasets with 7,000 cells or more, the median MAE is close to zero for both uniform and normal distributions at any dropout rate. In summary, scISR (using hypergeometric test) performs well for large datasets with high dropout rates even when the dropout probability is not uniformly distributed. Moreover, similar to dataset with 1,000 cells, scISR also outperforms other methods in recovering the missing values in bigger datasets.

Next, we investigate performance of scISR using simulated datasets in which the cells of the same cell type have high correlation. Denote m as the number of genes. We first generated four different vectors: i) $(\frac{0}{m}, \frac{1}{m}, \ldots, \frac{m}{m})$, ii) $(\frac{0}{m}, -\frac{1}{m}, \ldots, -\frac{m}{m})$ iii) $(\frac{0}{m}, \frac{2}{m}, \ldots, \frac{m}{m}, \frac{0}{m}, \frac{2}{m}, \ldots, \frac{m}{m})$, and iv) $(\frac{0}{m}, -\frac{2}{m}, \ldots, -\frac{m}{m}, \frac{0}{m}, -\frac{2}{m}, \ldots, -\frac{m}{m})$. Each vector was used to simulated a cell type. Instead of shifting the mean of a cell type, we added the first vector to the expression of the first cell type. Similarly, we added the second, third, and fourth vectors to the second, third, and fourth cell types, respectively. By doing so, cells of the same type will have high correlation. Similar to the above simulation, we added dropouts with various rates (60%, 70%, 80%, and 90%) and distributions (uniform and normal). We also simulated datasets of different numbers of cells: 1,000, 3,000, 5,000, 7,000, and 10,000.

Figure S20 shows the results obtained from the 40 new simulated datasets. We also used the same metrics to assess the similarity between imputed and the complete data: (1) mean absolute error (the smaller the better), and (2) correlation (the higher the better). scISR outperforms other methods by having the lowest mean absolute errors and highest correlations in every analysis performed.

To measure the accuracy of the hyper-geometric test as a standalone module, we compared the altered zero values against the ground truth (in which we know which zero is true zero and which is dropout). We define the following terms: 1) TP (a dropout value is altered by scISR), 2) FN (a dropout value not altered), 3) FP (a true zero value altered), and 4) TN (a true zero value not altered). For assessment purpose, we used the F-score to measure the accuracy of the hypothesis testing. Note that F-score is calculated based on precision and recall: $F1 = 2 \times \frac{precision \times recall}{precision + recall}$ or $F1 = \frac{TP}{TP + \frac{1}{2}(FP+FN)}$. In the ideal case, F-score equals to 1 if both precision and recall equal to 1 (i.e., $FP = FN = 0$). Figure S21 shows the F-score values obtained from the 40 simulated datasets (5 cell numbers $\times$ 4 dropout rates $\times$ 2 distributions). When the dropout probability is uniformly distributed, the median F-scores are close to 1 in all settings. When the dropout probability is normally distributed, the median values are less than 1 for small datasets with high dropout rates. However, as the sample size increases, the results improve. For datasets with 7,000 cells or more, the median F-scores are close to 1 for both uniform and normal distributions.

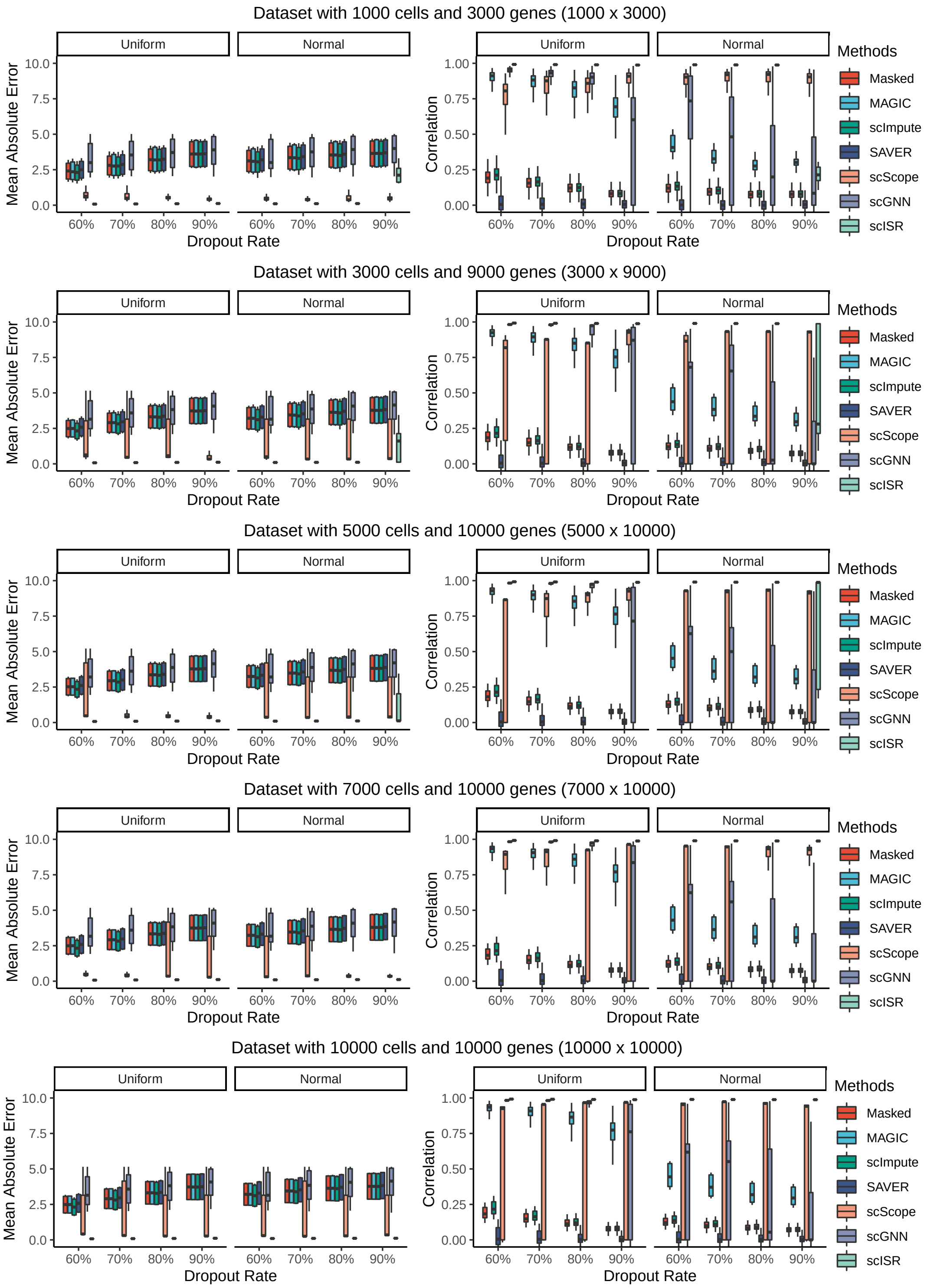

Figure S19. Assessment of MAGIC, scImpute, SAVER, scScope, scGNN, and scISR using simulated datasets with different dropout distributions and sample sizes. The left panels show the Mean Absolute Error (MAE) values while the right panels show the correlation coefficients. In each panel, the left side shows the results for uniform distributions while the right side shows the results for normal distributions. For small datasets (e.g., datasets with 1,000 cells) with high dropout rates, scISR is less accurate when the dropout probability is normally distributed. When the sample size increases, scISR becomes more accurate. For datasets with 7,000 cells or more, scISR performs well for both uniform and normal distributions alike across all dropout rates. For most of the dataset sizes and dropout rates, scISR have a much better median MAE and correlation compared to other methods.

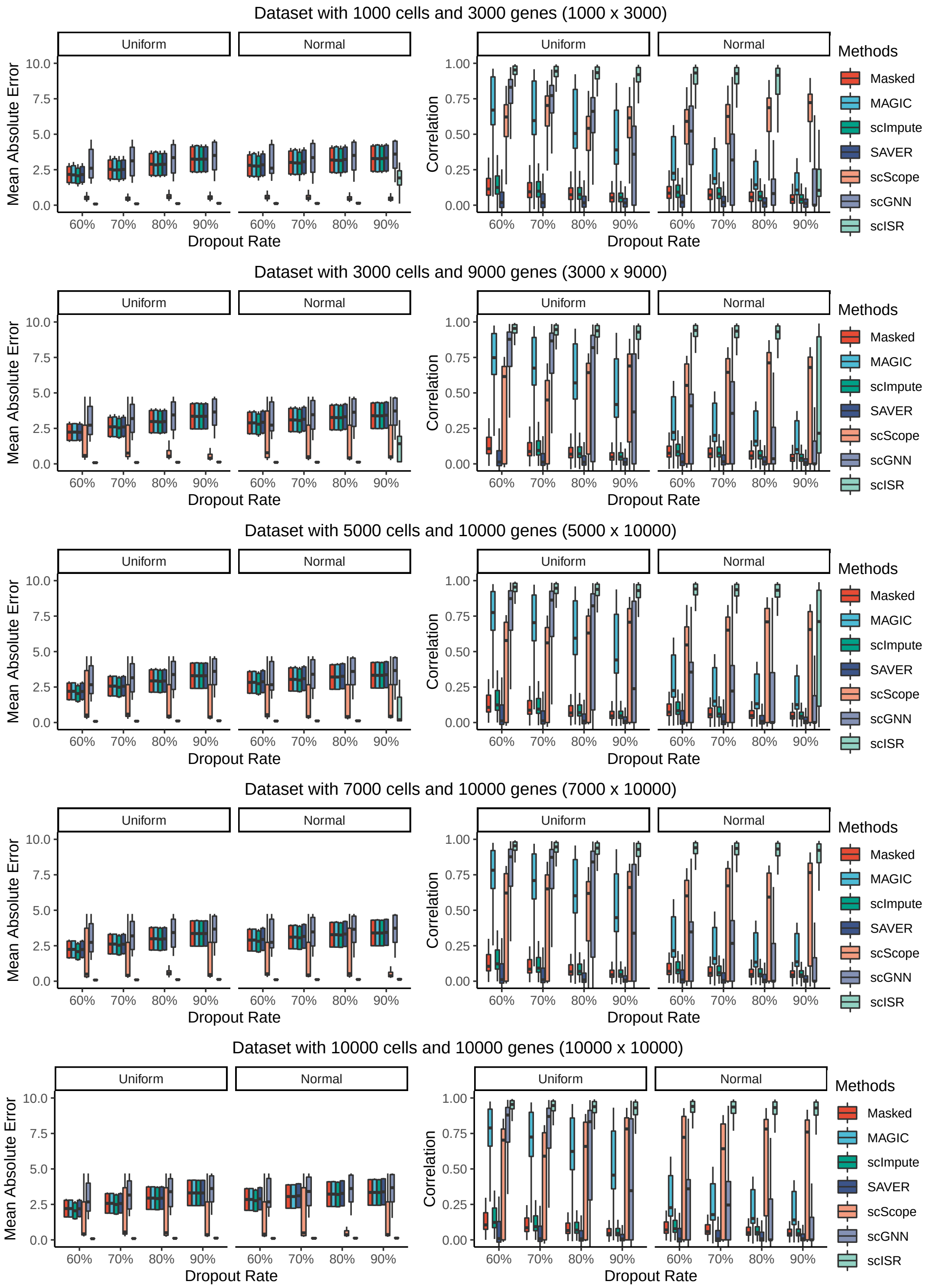

Figure S20. Assessment of MAGIC, scImpute, SAVER, scScope, scGNN, and scISR using simulated datasets with different dropout distributions and sample sizes. In each dataset, cells of the same type have high correlation and cells of different types have low correlation. The left panels show the Mean Absolute Error (MAE) values while the right panels show the correlation coefficients. In each panel, the left side shows the results for uniform distributions while the right side shows the results for normal distributions. For small datasets (e.g., datasets with 1,000 cells) with high dropout rates, scISR is less accurate when the dropout probability is normally distributed. When the sample size increases, scISR becomes more accurate. For datasets with 7,000 cells or more, scISR performs well for both uniform and normal distributions alike across all dropout rates. For most of the dataset sizes and dropout rates, scISR have a much better median MAE and correlation compared to other methods.

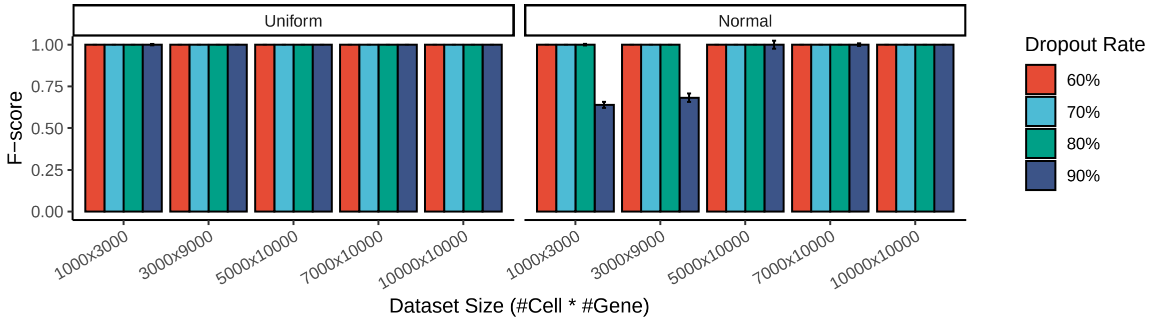

Figure S21. The accuracy of scISR hypothesis testing using F-score. The F-score measures how well the algorithm distinguish between true zero values and dropouts. The left panel shows the F-scores for datasets with uniform distribution while the right panel shows the F-scores for datasets with normal distribution. For datasets with 7,000 cells or more, the median F-scores are close to 1 for both uniform and normal distributions alike across all dropout rates. In other words, scISR accurately identifies the zero values that need to be imputed.

4.3 Simulation studies using Splatter package

Using Splatter R package[16], we perform additional simulation with negative binomial distribution as noise model. We set the number of genes to 15,000 and the number of cell types to 3. We generated 30 datasets with different cell numbers: 5,000, 10,000, 25,000, 50,000, 100,000 and 200,000. For each sample size, we varied the sparsity levels by adjusting the *dropout.mid* parameters (midpoint parameter for dropout logistic function of Splatter). We set *dropout.mid* to 2.5, 3, 3.5, 4, and 4.5, which led to sparsity levels of 84%, 87%, 89%, 91%, 93%, respectively. Both sample size (hundreds of thousands of cells) and dropout rates (84%–93%) are often expected from current scRNA-seq datasets. In total, we simulated 30 new datasets using Splatter (6 cell numbers $\times$ 5 sparsity levels).

We used the mean absolute error (MAE) values and correlation coefficients between the ground truth expression and imputed expression data to assess the performance of imputation methods. Figure S22 shows the results, in which scISR and scScope are the only methods that can perform imputation on the biggest dataset. MAGIC, SAVER, scImpute, and scGNN cannot analyze datasets with are more than 100,000, 10,000, 10,000, and 50,000 cells, respectively. For large datasets, these methods either returned error, ran out of memory (the memory limit on our machine is 128 GB), or could not finish the analysis in a reasonable amount of time (more than one day).

Overall, MAGIC, SAVER, scScope, and scGNN are unable to correctly recover the missing values, which leads to MAE values that are even higher than the masked data (data without imputation). scImpute has good results in small datasets but is unable to impute datasets with more than 10,000 cells. Even in datasets with 10,000 cells, scImpute returns errors when the dropout rate increases (91% and 93%). In contrast, scISR is able to improve the quality of the dropout data in all scenarios.

We also report the running time for these simulation studies. As seen in Figure S23, scISR and scScope are the only methods that can perform imputation on dataset with 200,000 cells. The reason scScope can analyze the biggest dataset in this simulation is because the number of genes is set to 15,000, which is lower than that of real datasets. Both methods can analyze the largest dataset with 200,000 cells in approximately 100 to 200 minutes. Other methods either run out of memory or are unable to finish in a reasonable amount of time, which was set to one day.

4.4 Robustness of scISR against batch effect

To investigate the effect of batch effect on scISR, we tested our approach using simulated datasets generated by Splatter package. We used the following parameters: the number of genes is set to 15,000; 5 sparsity levels are generated with zero ratios ranging from 84% to 93%; the number of cells is fixed to 25,000; batch effect is either enable or disable. Splatter simulates batch effect by generating a small scaling factor for each gene in each batch. We generated a total of 10 datasets using these parameters. As seen in Figure S24, batch effects do not have a significant impact on the performance of scISR.

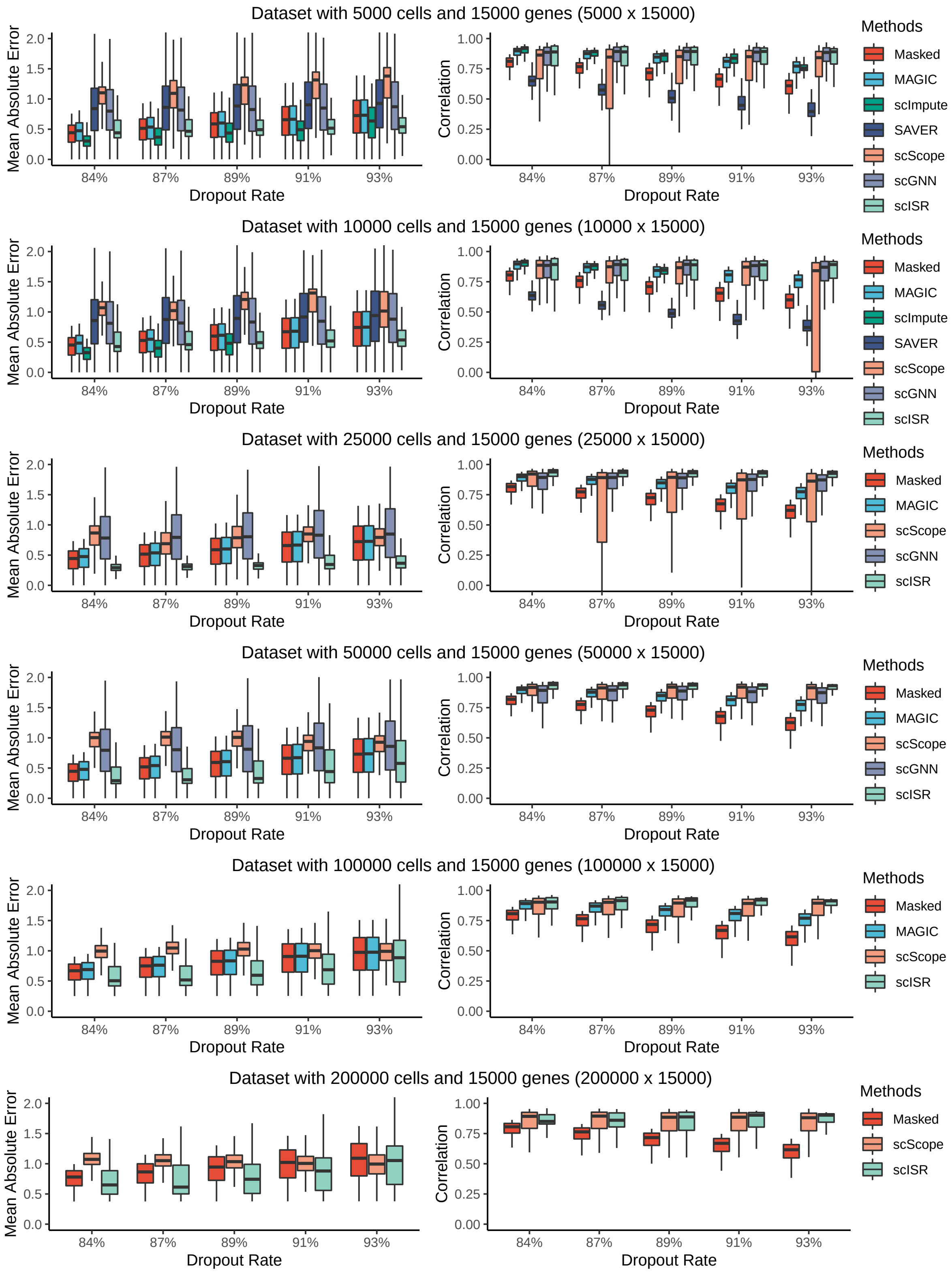

Figure S22. Assessment of MAGIC, scImpute, SAVER, scScope, scGNN, and scISR using datasets simulated by Splatter. The left panels show the Mean Absolute Error (MAE) values while the right panels show the correlation coefficients. scISR and scScope are the only methods that can perform imputation on the biggest dataset, while MAGIC, SAVER, scImpute, and scGNN stop working with datasets bigger than 100,000, 10,000, 10,000, and 50,000 cells, respectively. scISR is the only method that can improve the dropout data in all scenarios.

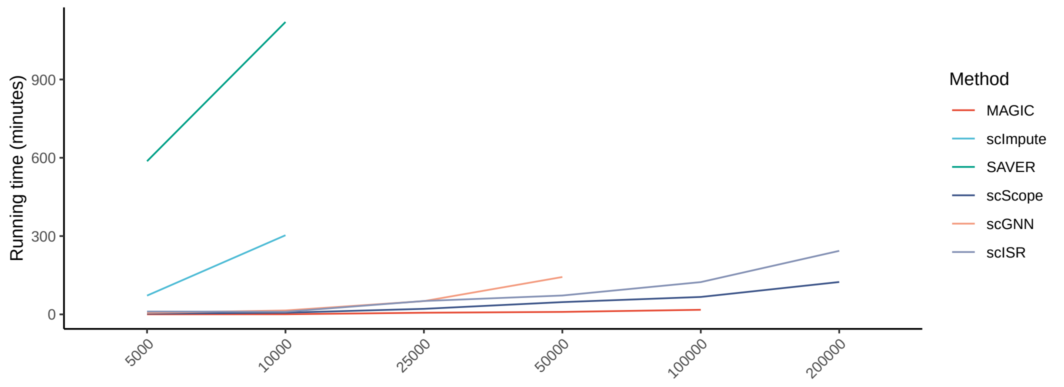

Figure S23. Running time of the six imputation methods on simulated datasets. These datasets have 15,000 cells and varying number of cells (5,000 to 200,000). scISR and scScope are the only methods that can analyze all datasets. The two methods can finish the analysis of 200,000 cells in 200 and 100 minutes, respectively.

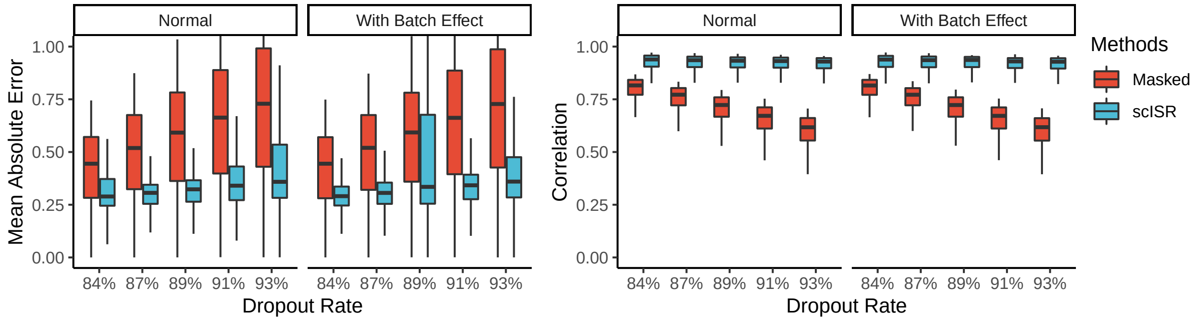

Figure S24. Impact of batch effects on scISR. The left panels show the Mean Absolute Error (MAE) values while the right panels show the correlation coefficients. In each panel, the left 10 boxes show the results for data without batch effects while the right 10 boxes show the results for data with batch effects. Overall, batch effects do not have a significant impact on the performance of scISR.

References

1. Barrett, T. *et al.* NCBI GEO: archive for functional genomics data sets–update. *Nucleic Acids Research* **41**, D991–D995 (2013).
2. Rustici, G. *et al.* ArrayExpress update–trends in database growth and links to data analysis tools. *Nucleic Acids Research* **41**, D987–D990 (2013).
3. Van Dijk, D. *et al.* Recovering gene interactions from single-cell data using data diffusion. *Cell* **174**, 716–729 (2018).
4. Li, W. V. & Li, J. J. An accurate and robust imputation method scImpute for single-cell RNA-seq data. *Nature Communications* **9**, 997 (2018).
5. Huang, M. *et al.* SAVER: gene expression recovery for single-cell RNA sequencing. *Nature Methods* **15**, 539–542 (2018).
6. Deng, Y., Bao, F., Dai, Q., Wu, L. F. & Altschuler, S. J. Scalable analysis of cell-type composition from single-cell transcriptomics using deep recurrent learning. *Nature Methods* **16**, 311–314 (2019).
7. Wang, J. *et al.* scgnn is a novel graph neural network framework for single-cell rna-seq analyses. *Nature communications* **12**, 1–11 (2021).
8. Hubert, L. & Arabie, P. Comparing partitions. *Journal of Classification* **2**, 193–218 (1985).
9. Jaccard, P. Étude comparative de la distribution florale dans une portion des Alpes et des jura. *Bull Soc Vaudoise Sci Nat* **37**, 547–579 (1901).
10. Manning, C., Raghavan, P. & Schütze, H. Introduction to information retrieval. *Natural Language Engineering* **16**, 100–103 (2010).
11. Scrucca, L., Fop, M., Murphy, T. B. & Raftery, A. E. mclust 5: clustering, classification and density estimation using Gaussian finite mixture models. *The R Journal* **8**, 205–233 (2016).
12. van der Maaten, L. & Hinton, G. Visualizing data using t-SNE. *Journal of Machine Learning Research* **9**, 2579–2605 (2008).
13. Becht, E. *et al.* Dimensionality reduction for visualizing single-cell data using UMAP. *Nature Biotechnology* **37**, 38–44 (2019).
14. Satija, R., Farrell, J. A., Gennert, D., Schier, A. F. & Regev, A. Spatial reconstruction of single-cell gene expression data. *Nature Biotechnology* **33**, 495–502 (2015).
15. Székely, G. J., Rizzo, M. L. & Bakirov, N. K. Measuring and testing dependence by correlation of distances. *The Annals of Statistics* **35**, 2769–2794 (2007).
16. Zappia, L., Phipson, B. & Oshlack, A. Splatter: Simulation of single-cell RNA sequencing data. *Genome Biology* **18**, 1–15 (2017).
